# DNA Stabilizes Eight-Electron Superatom Silver Nanoclusters with Broadband Downconversion and Microsecond-Lived Luminescence

**DOI:** 10.1021/acs.jpclett.2c02207

**Published:** 2022-08-29

**Authors:** Anna Gonzàlez-Rosell, Rweetuparna Guha, Cecilia Cerretani, Vanessa Rück, Mikkel B. Liisberg, Benjamin B. Katz, Tom Vosch, Stacy M. Copp

**Affiliations:** † Department of Materials Science and Engineering, University of California, Irvine, California 92697, United States; ‡ Nanoscience Center and Department of Chemistry, University of Copenhagen, Universitetsparken 5, 2100 Copenhagen, Denmark; § Department of Chemistry, University of California, Irvine, California 92697, United States; ∥ Department of Physics and Astronomy, University of California, Irvine, California 92697, United States; ⊥ Department of Chemical and Biomolecular Engineering, University of California, Irvine, California 92697, United States

## Abstract

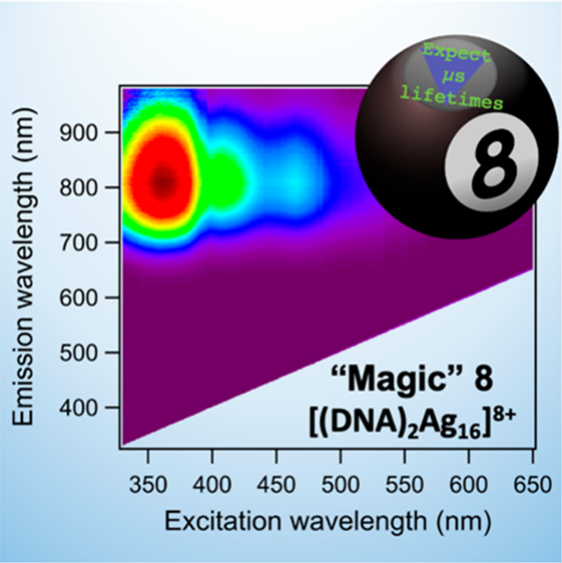

DNA oligomers are known to serve as stabilizing ligands for silver nanoclusters (Ag_*N*_-DNAs) with rod-like nanocluster geometries and nanosecond-lived fluorescence. Here, we report two Ag_N_-DNAs that possess distinctly different structural properties and are the first to exhibit only microsecond-lived luminescence. These emitters are characterized by significant broadband downconversion from the ultraviolet/visible to the near-infrared region. Circular dichroism spectroscopy shows that the structures of these two Ag_*N*_-DNAs differ significantly from previously reported Ag_*N*_-DNAs. We find that these nanoclusters contain eight valence electrons, making them the first reported DNA-stabilized luminescent quasi-spherical superatoms. This work demonstrates the important role that nanocluster composition and geometry play in dictating luminescence properties of Ag_*N*_-DNAs and significantly expands the space of structure–property relations that can be achieved for Ag_*N*_-DNAs.

DNA oligomers can serve as multidentate ligands to stabilize silver nanoclusters (Ag_*N*_-DNAs) with tunable optical properties. Silver cations (Ag^+^) interact with DNA through the nucleobases,^1^ giving Ag_*N*_-DNAs genomic-like properties.^2^ The nucleobase sequence encodes the number of silver atoms, *N*, and the size-correlated excitation and emission wavelengths of Ag_*N*_-DNAs. DNA strands of different sequences have yielded a diverse spectral palette of Ag_*N*_-DNAs with colors from 400 nm up to 1000 nm.^2^ In conjunction with high fluorescence quantum yields (QYs), large Stokes shifts as compared to organic dyes,^2,3^ and unique photophysics,^4,5^ Ag_*N*_-DNAs are promising emitters for sensing^6^ and biomedical imaging.^4,7,8^

Studies have demonstrated that the silver nanocluster cores of fluorescent Ag_*N*_-DNAs can be rod-like. In contrast to the spheroidal geometries typical of monolayer-protected Ag_*N*_,^9^ Schultz et al. proposed that DNA ligands impose highly anisotropic, rod-like geometries on the encapsulated Ag_*N*_.^10^ This prediction was supported by other studies^11−13^ and confirmed by the first published crystal structures of fluorescent Ag_*N*_-DNAs.^14,15^ High-resolution electrospray ionization mass spectrometry^10^ (HR-ESI-MS) studies show that fluorescence excitation and emission wavelengths of Ag_*N*_-DNAs are correlated with the numbers of effective valence electrons within the nanocluster core and that fluorescent Ag_*N*_-DNAs have a preference for even numbers of valence electrons.^12^ Because silver has a valency of 1, the valence electron count is often described in the literature as the Ag^0^ content of the nanocluster, *N*_0_, rather than total silver content *N*.^2^ Ag_*N*_-DNAs with green and red fluorescence possess “magic numbers” of *N*_0_ = 4 and 6, respectively,^12^ and a few near-infrared (NIR) fluorescent Ag_*N*_-DNAs with emission peaks between 775 and 1000 nm are known to contain *N*_0_ = 10 and 12.^12,16^ No values of *N*_0_ have been reported so far for Ag_*N*_-DNAs with peak emission between 685 and 775 nm. Notably, the *N*_0_ = 4, 6, 10, and 12 of fluorescent Ag_*N*_-DNAs differ from the “superatomic” magic numbers for quasi-spherical ligand-stabilized nanoclusters (*N*_0_ = 2, 8, ...).^17^ To date, no fluorescent Ag_*N*_-DNAs have been reported with *N*_0_ = 2 or 8, and only two nonfluorescent Ag_*N*_-DNAs are reported to contain *N*_0_ = 8.^12^ The eight-electron “magic” monolayer-protected gold and silver nanoclusters are highly prevalent and correspond to a 1S^2^ 1P^6^ superatomic electron configuration.^18,19^ The low abundance of eight-electron Ag_*N*_-DNAs may indicate that DNA ligands prefer to stabilize nanoclusters without spherical symmetry.

Here, we report the first evidence for luminescent DNA-stabilized eight-electron Ag_*N*_, which possess distinctly different structures and photophysical properties than previously reported Ag_*N*_-DNAs. Optical and compositional characterization of two HPLC-purified NIR-emissive Ag_*N*_-DNAs with atypical multipeaked excitation spectra and chiroptical signatures are presented. HR-ESI-MS shows that these Ag_*N*_-DNAs possess *N*_0_ = 8 valence electrons. These Ag_*N*_-DNAs have no significant nanosecond-lived fluorescence but instead present microsecond-lived luminescence and larger broadband downconversion than any other Ag_*N*_-DNAs reported to date.^20^ Our findings support that the investigated *N*_0_ = 8 Ag_*N*_-DNAs have distinctly different compositions and geometries compared to previously reported rod-shaped Ag_*N*_-DNAs. These results demonstrate the influence of nanocluster composition and geometry on the luminescence properties of nanocluster emitters.

The Ag_*N*_-DNAs studied here were identified using high-throughput synthesis and characterization methods.^12^ The DNA oligomer 5′-ATCTCCACAG-3′ stabilizes a species with an emission peak at 800 nm (800-Ag_*N*_-DNA), reported by Swasey et al.^16^ The oligomer 5′-GACGACGGAT-3′ stabilizes a species with peak emission at 760 nm (760-Ag_*N*_-DNA), reported in a large-scale study of Ag_*N*_-DNA excitation and emission spectra by Copp et al.^3^ Samples were purified by high performance liquid chromatography (HPLC) prior to characterization to ensure the presence of only one emissive species in solution (Figures S1–S4).

Figure 1 shows that 800-Ag_*N*_-DNA and 760-Ag_*N*_-DNA possess absorption and excitation spectra that are uncharacteristically complex as compared to other HPLC-purified Ag_*N*_-DNAs,^2^ motivating further study. The absorption spectra of these Ag_*N*_-DNAs exhibit several distinct bands corresponding to the nanocluster at near-UV and blue wavelengths (Figure 1A,C), along with the absorption and excitation features around 260 nm that are due to UV transitions of the nucleobases.^21^ 800-Ag_*N*_-DNA exhibits a dominant Ag_*N*_-related absorbance peak at 370 nm, with two less intense yet defined peaks at 410 and 460 nm, and a tail that spans from 500 to 570 nm. 760-Ag_*N*_-DNA exhibits two dominant absorbance peaks at 355 nm (with a shoulder around 335 nm) and 455 nm and minor features at 405, 495, and 575 nm. 2D emission vs excitation maps for these Ag_*N*_-DNAs (Figure 1B,D) show that the emission spectrum is independent of the excitation wavelength (see also Figure S4), supporting the presence of only one emissive species in solution. This is also confirmed by the good overlap between the absorption and excitation spectra of both 800-Ag_*N*_-DNA and 760-Ag_*N*_-DNA (Figure S3).

**Figure 1:**
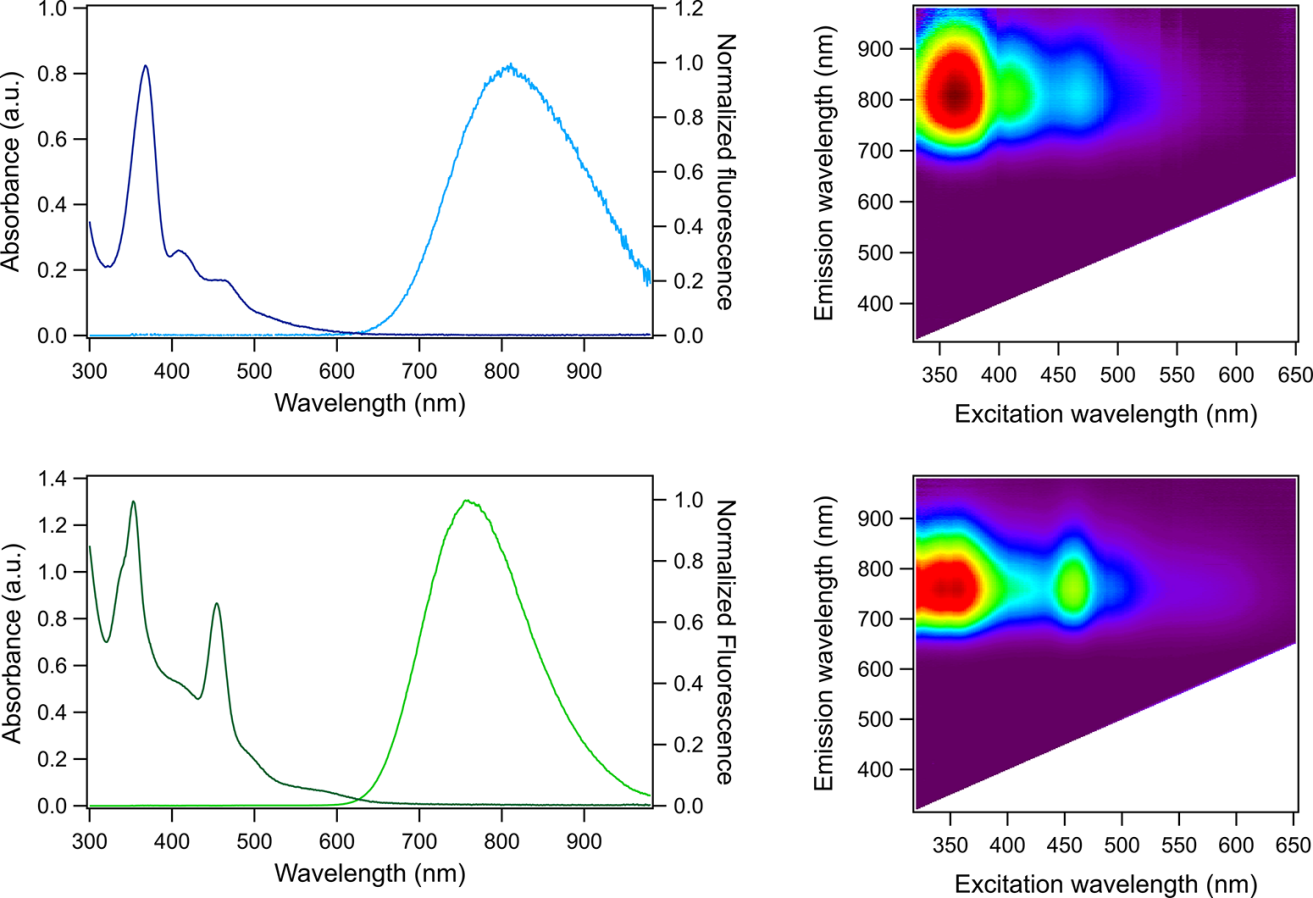
(A) Normalized absorbance (dark blue) and normalized emission (light blue) spectra and (B) 2D excitation/emission plot for 800-Ag_*N*_-DNA. (C) Absorbance (dark green) and emission (light green) spectra and (D) 2D emission vs excitation plot for 760-Ag_*N*_-DNA. All measurements were conducted in 10 mM NH_4_OAc, recorded at room temperature. Emission spectra in (A) and (C) were recorded using a UV LED excitation source.

While small excitation features at short wavelengths have been reported for other NIR-emissive Ag_*N*_-DNAs, all previously reported HPLC-purified Ag_*N*_-DNAs in the literature have one intense excitation peak in the green to NIR spectral region.^20,22,23^ The simplicity of typical Ag_*N*_-DNA excitation spectra contrasts with the multipeaked absorbance spectra of monolayer-protected Ag_*N*_.^19^ Notably, 800-Ag_*N*_-DNA and 760-Ag_*N*_-DNA share excitation/absorption features with other eight-electron Ag_*N*_.^24−26^ Similar features are also observed for zeolite-stabilized Ag_*N*_,^27,28^ which exhibit strong excitation peaks in the 250 to 450 nm region with broad downconversion to the visible/NIR range.

800-Ag_*N*_-DNA and 760-Ag_*N*_-DNA have room temperature (luminescence) QYs of 1% and 4%, respectively, which are lower than those reported for other NIR-emissive Ag_*N*_-DNAs.^10,23,29^ As a result of the significant difference between excitation and emission wavelengths of these nanoclusters, the QY was determined using 736-Ag_16_-DNA, a well-studied NIR Ag_*N*_-DNA with an unusually large Stokes shift, as the reference compound (Figures S5 and S6).^15,20,30^

Electronic circular dichroism (CD) spectra of 800-Ag_*N*_-DNA and 760-Ag_*N*_-DNA were compared to those of four previously reported HPLC-purified Ag_*N*_-DNAs.^31^ CD spectroscopy, which measures the difference in absorbance of left and right circularly polarized light, is highly sensitive to the electronic structure. DNA is an inherently chiral biomolecule with electronic transitions below ∼300 nm, and changes in middle UV (200–300 nm) CD signatures indicate DNA conformational changes. Swasey et al. showed that adding Ag^+^ to DNA alters the middle UV CD spectrum as compared to bare DNA, demonstrating Ag^+^-mediated DNA rearrangement. Reduction of the Ag^+^-DNA complex forms Ag_*N*_-DNAs, and new CD features arise at near-UV (300–400 nm) and visible to NIR wavelengths as a result of the effective valence electrons of the nanoclusters.^31,32^ All previously reported CD spectra of Ag_*N*_-DNAs show strong negative signals in the near-UV region and a positive peak in the visible-NIR region that is aligned with the dominant absorbance peak of the Ag_*N*_-DNA.^31^

The CD spectra for 800-Ag_*N*_-DNA and 760-Ag_*N*_-DNA (Figure 2) differ from these past studies in several ways. First, both negative *and* intense positive CD features are present from 200 to 300 nm, supporting different DNA ligand conformations. CD transitions > 300 nm, which are attributed to the Ag_*N*_,^31^ also differ from past reported behavior. For 800-Ag_*N*_-DNA (Figure 2A), the dominant CD feature at 370 nm is bisignate, indicating the presence of two separate transitions. This may explain why the absorbance peak at 370 nm is not perfectly symmetrical (Figure 1A). Additional longer wavelength CD features present a negative Cotton effect, in contrast to previously reported Ag_*N*_-DNAs.^31^ We observe similar behavior for 760-Ag_*N*_-DNA (Figure 2B), with bisignate transitions between 350 and 400 nm and negative visible peaks. The clear differences between CD spectra of 800-Ag_*N*_-DNA and 760-Ag_*N*_-DNA compared to previously studied Ag_*N*_-DNAs suggest significant structural differences between these two classes of nanoclusters.

**Figure 2:**
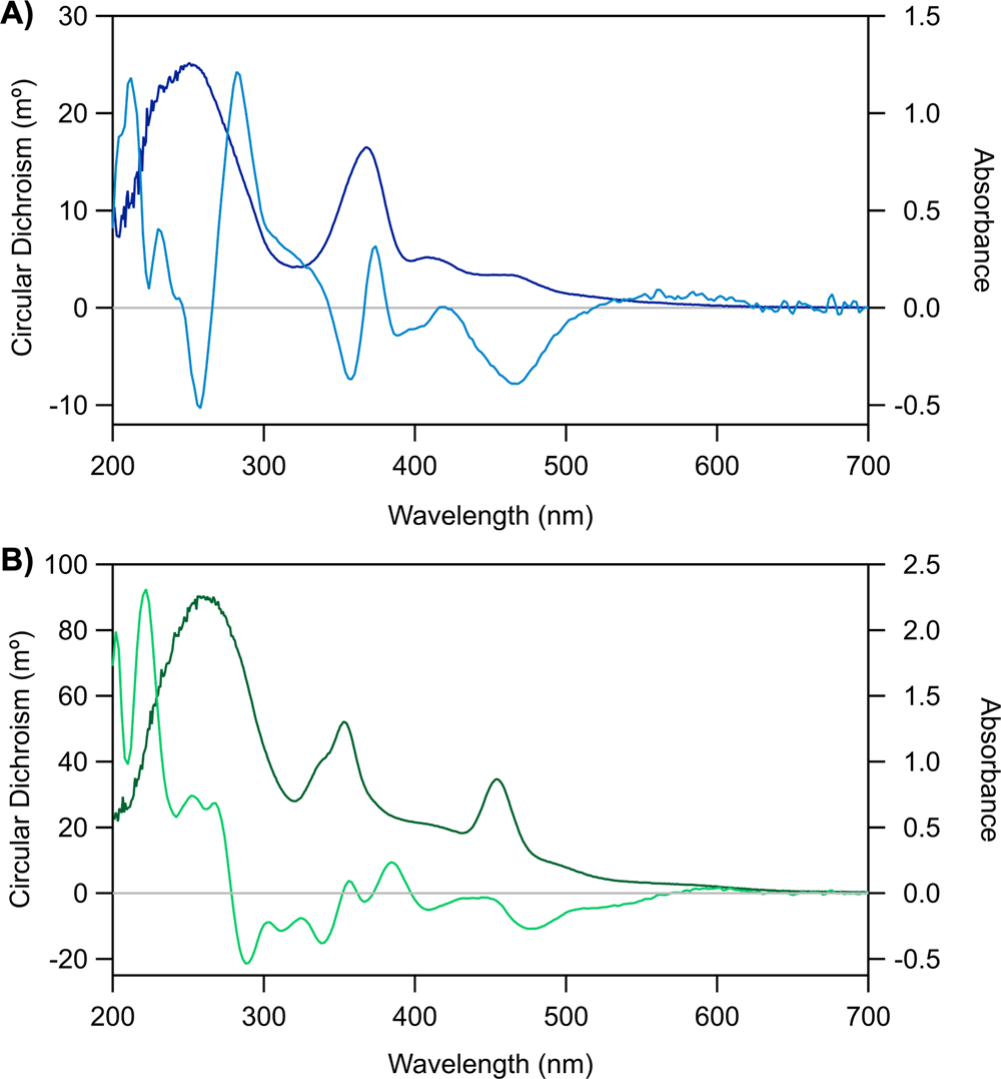
CD spectra (light blue/green) and absorbance spectra (dark blue/green) of (A) 800-Ag_*N*_-DNA and (B) 760-Ag_*N*_-DNA in 10 mM NH_4_OAc at room temperature (see SI for details).

We determined the compositions and total charges of 800-Ag_*N*_-DNA and 760-Ag_*N*_-DNA by negative ion mode HR-ESI-MS, which is well-suited for characterizing noncovalent interactions in nucleic acids.^33^ HR-ESI-MS allows determination of both ion mass, *m*, and charge, *z*, rather than just the ratio *m*/*z*, by resolving the isotope pattern that arises due to the natural variation in isotopic abundances of the elements.^10,34^ By this method, we not only count the number of silver atoms, *N*, and DNA strands, *n*_*s*_, but also separate *N* into effective neutral (*N*_0_) and cationic (*N*_*+*_) silver content, thereby determining the oxidation state of the Ag_*N*_-DNA and the effective valence electron count equivalent to *N*_0_.^10^

Mass spectra are shown in Figure 3. Both Ag_*N*_-DNAs show dominant products for charge states *z* = 4^–^ and *z* = 5^–^. For 800-Ag_*N*_-DNA, the most intense peak at each charge state (*z* = 4^–^ in Figure 3A; *z* = 5^–^ in Figure S8) corresponds to a product composed of two DNA strands (*n*_*s*_ = 2) and *N* = 16 total silvers, with an overall charge of +8, i.e., [(DNA)_2_Ag_16_]^8+^ (see Table S1 for fits). Thus, this Ag_*N*_-DNA has eight effective valence electrons, *N*_0_ = 8. Two peaks with smaller intensities at each charge state correspond to *n*_*s*_ = 2 with *N* = 13 and 14, respectively, and preserve the same value of *N*_0_ = 8 (Figures S7 and S9). For 760-Ag_*N*_-DNA (Figure 3B and Figure S10), the two most intense peaks correspond to a product of *n*_*s*_ = 2 and *N* = 17, with an overall nanocluster charge of +9, i.e., [(DNA)_2_Ag_17_]^9+^. Thus, this nanocluster also has *N*_0_ = 8. Minor peaks correspond to smaller *n*_*s*_ = 2 products with *N* = 10 to 16 and different overall nanocluster charges (Figure S7). Because samples were HPLC-purified prior to characterization, the smaller products likely form due to loss of silvers during the electrospray process, as is common for other Ag_*N*_-DNAs.^16^ Because *N*_0_ is conserved in the dominant products at *z* = 4^–^ and *z* = 5^–^ charge states for both emitters (Figures S8 and S10 and Table S1), these results support that the nanocluster cores of 800-Ag_*N*_-DNA and 760-Ag_*N*_-DNA host eight effective valence electrons. (Note that Ag_N_-DNA peaks are distinctly resolved from adducts of additional Na^+^ cations, Figure S11.) These are the first reported luminescent Ag_N_-DNAs with *N*_0_ = 8, a “magic number” for spherical gold and silver superatoms.^26,35^

**Figure 3:**
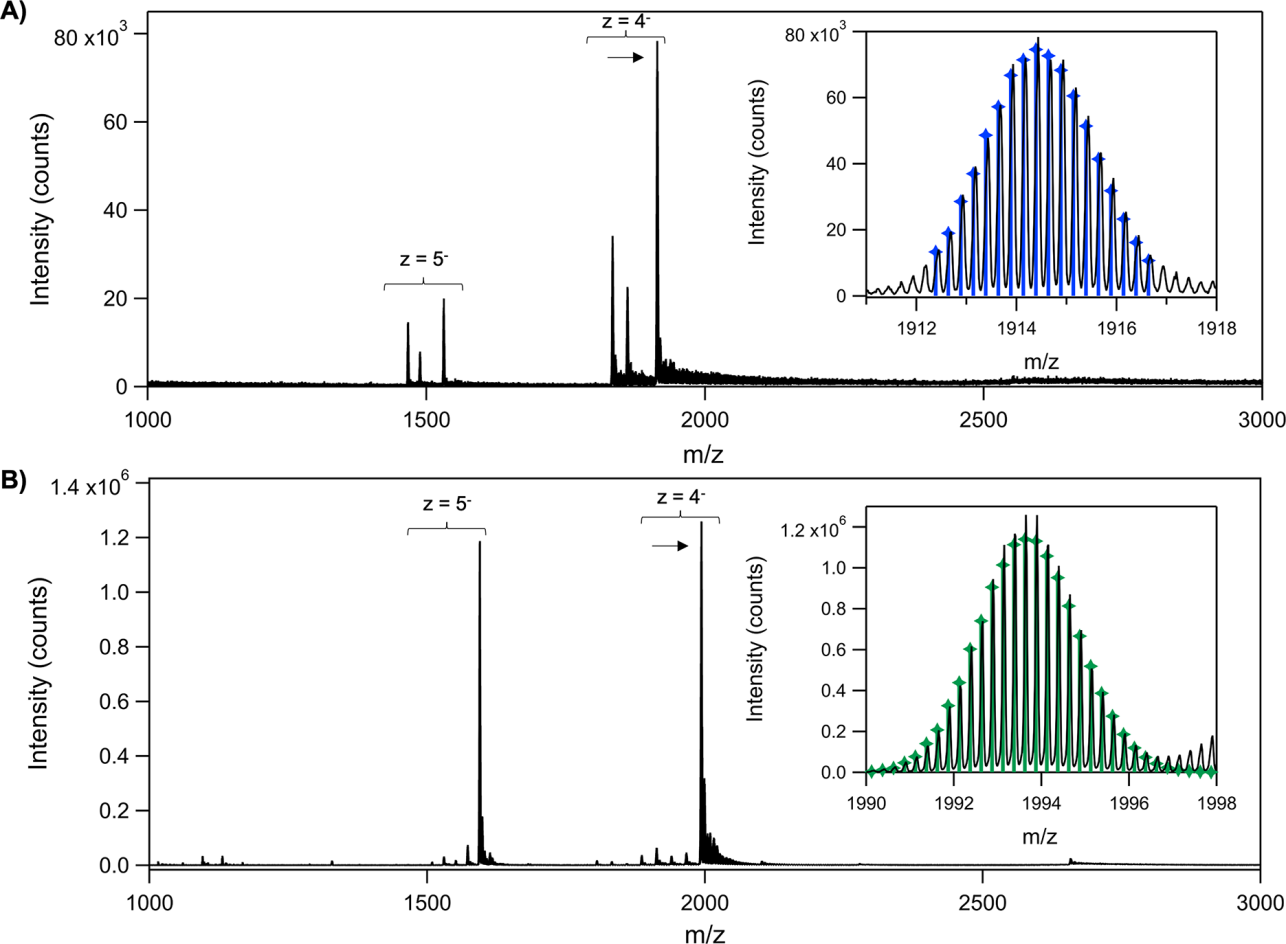
Mass spectra of (A) 800-Ag_*N*_-DNA and (B) 760-Ag_*N*_-DNA. Experimental data in black. Arrows indicate the experimental peaks shown in insets, with calculated isotopic distributions for (A) [(DNA)_2_Ag_16_]^8+^ (blue) and (B) [(DNA)_2_Ag_17_]^9+^ (green) at *z* = 4^–^.

The optical spectra, lower QYs, and compositions of 800-Ag_*N*_-DNA and 760-Ag_*N*_-DNA support that these emitters have clear structural differences compared to previously reported fluorescent Ag_*N*_-DNAs. These differences may influence their photophysical properties. Hence, we investigated the nature of the luminescence process in *N*_0_ = 8 Ag_N_-DNAs using a burst mode approach.^36^ Both Ag_*N*_-DNAs only show evidence for microsecond-lived luminescence (Figure 4) at room temperature. For 800-Ag_*N*_-DNA, the intensity decay upon turning off the laser was tail-fitted monoexponentially, resulting in a decay time of 2.39 ± 0.01 μs. The decay curve of 760-Ag_*N*_-DNA was tail-fitted with a monoexponential model, resulting in a decay time of 19.5 ± 0.1 μs. These values are in good agreement with the decay times obtained using time-correlated single photon counting (TCSPC) and a Xenon flash lamp as an excitation source (Figures S12 and S13).

**Figure 4:**
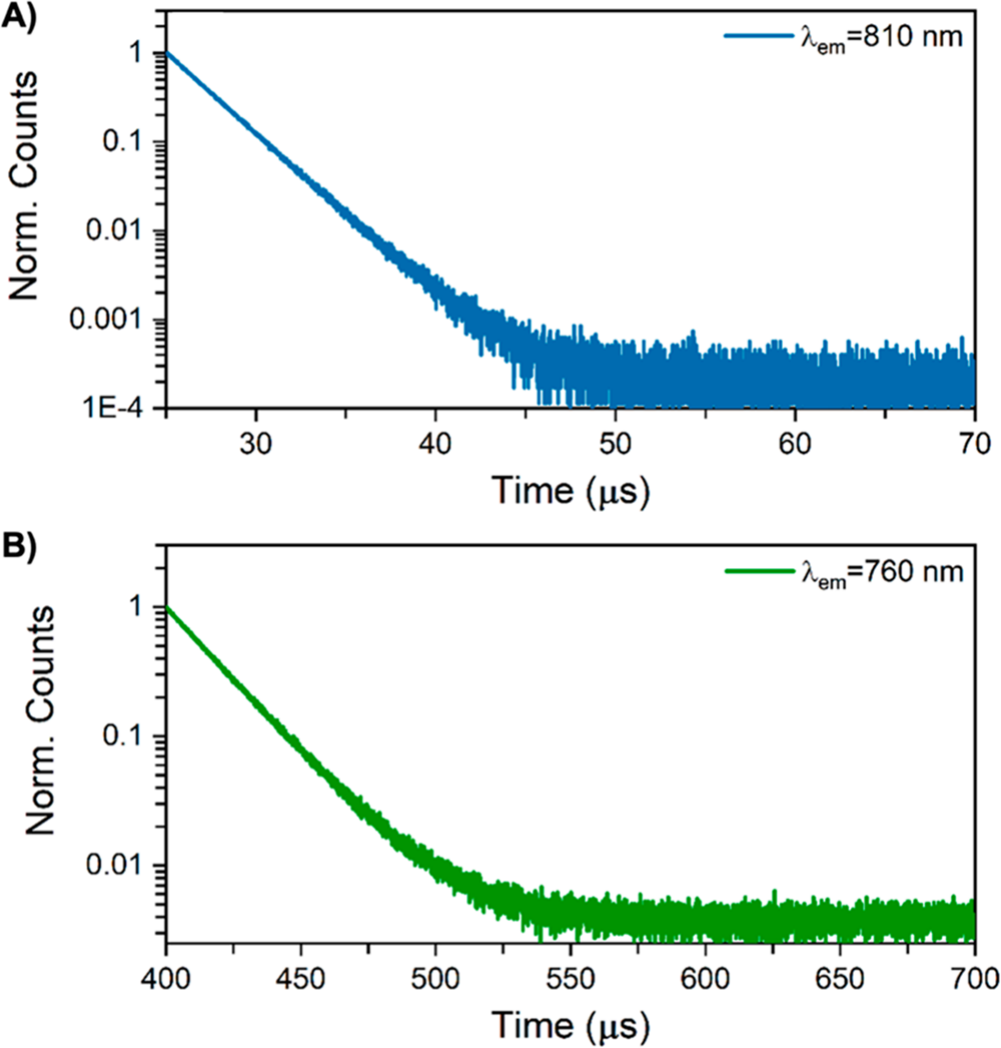
Decay curves of (A) 800-Ag_*N*_-DNA and (B) 760-Ag_*N*_-DNA measured in burst mode, exciting at 374.3 nm with a picosecond-pulsed laser (PicoQuant). Measurements were carried out at 25 °C in 10 mM NH_4_OAc. The full burst mode data, together with the corresponding instrument response functions (IRFs) and monoexponential fits, can be found in Figures S14 and S15.

Microsecond-lived luminescence is usually indicative of a spin-forbidden phosphorescence process, which is sensitive to quenching by Dexter energy transfer with molecular oxygen. O_2_ has a triplet ground state and is widely used to quench phosphorescence from organic molecules. We investigated the effect of O_2_ on the decay time of 800-Ag_*N*_-DNA to determine if the microsecond-lived luminescence of this emitter is phosphorescence-like. The concentration of dissolved O_2_ was lowered in the 800-Ag_*N*_-DNA solution using a water jet pump, and no change in the microsecond lifetime was observed (Figure S16, Table S3). The absence of an effect on the microsecond lifetime could indicate a nonphosphorescence-based decay pathway or might be caused by the DNA scaffold acting as a barrier that prevents the diffusion of O_2_ toward the Ag_*N*_ cluster. Given the remaining uncertainty, we prefer to refer to the microsecond-lived radiative emission as luminescence instead of phosphorescence for these Ag_*N*_-DNAs.

Microsecond-lived NIR luminescence was first reported by Rück et al. for an HPLC-purified Ag_*N*_-DNA that also exhibited green nanosecond fluorescence.^22^ Similarly, Petty et al. reported an unpurified Ag_*N*_-DNA with dual green fluorescence and NIR phosphorescence.^37^ In contrast, 800-Ag_*N*_-DNA and 760-Ag_*N*_-DNA show only microsecond-lived luminescence with no significant nanosecond-lived fluorescence. This suggests that the electronic structures of 800-Ag_*N*_-DNA and 760-Ag_*N*_-DNA differ from most previously investigated Ag_*N*_-DNAs characterized by nanosecond-lived fluorescence.^38^

As a result of the similarities among the eight-electron Ag_*N*_-DNAs reported here and other ligand-protected eight-electron superatom Ag_*N*_,^24−26^ we propose that 800-Ag_*N*_-DNA and 760-Ag_*N*_-DNA have quasi-spherical cluster geometries. Purified emissive Ag_N_-DNAs whose *N*_0_ have been determined by HR-ESI-MS^10,12,16,39^ have single dominant visible to NIR absorption peaks that are well-described by calculations for linear atomic chains of Ag atoms.^10,40^ In contrast, the most intense absorption peaks of 800-Ag_*N*_-DNA and 760-Ag_*N*_-DNA are at UV energies (Figure 1). Calculations show that quasi-spherical or globular Ag_N_ have intense cluster-related absorption peaks around 3.5 eV or higher,^41,42^ which suggests that 800-Ag_*N*_-DNA and 760-Ag_*N*_-DNA may be more quasi-spherical and less rod-like than fluorescent Ag_N_-DNAs. The distinct photophysical differences between rod-like Ag_*N*_-DNAs with nanosecond-lived fluorescence and eight-electron superatom Ag_*N*_-DNAs with microsecond-lived luminescence illustrate how nanocluster composition and shape influence the electronic structure of Ag_*N*_-DNAs. Gold nanoclusters also exhibit shape-dependent photophysics.^43^ For example, spherical Au_25_(SR)_18_^–^ exhibits ∼100 ns luminescence lifetimes, while rod-shaped [Au_25_(PPh_3_)_10_(SR)_5_Cl_2_]^2+^ display microsecond luminescence decay times and distinct absorbance spectra compared to Au_25_(SR)_18_^–^. Interestingly, we find that Ag_*N*_-DNAs exhibit opposite lifetime behaviors, with nanosecond-scale fluorescence for rod-like Ag_*N*_-DNAs and microsecond-scale luminescence for eight-electron Ag_*N*_-DNAs. Future structural characterization and computational studies are needed to confirm the hypothesis that the eight-electron Ag_*N*_-DNAs reported here possess spherical geometry and to better understand the structure–property correlations of Ag_*N*_-DNAs in general.

In conclusion, we present two HPLC-purified NIR-emissive Ag_*N*_-DNAs with broadband UV/vis to NIR downconversion, microsecond-lived luminescence, and *N*_0_ = 8 effective valence electrons in the Ag_*N*_ core. These are the first Ag_*N*_-DNAs reported with only microsecond-lived luminescence but no concurrent nanosecond fluorescence. These are also the first reported luminescent Ag_*N*_-DNAs with *N*_0_ = 8 superatomic shell filling numbers. The distinct photophysical and chiroptical properties of these Ag_*N*_-DNAs, as compared to previously investigated Ag_*N*_-DNAs, together with their eight effective valence electrons, support that these nanoclusters may be spherical unlike fluorescent Ag_*N*_-DNAs, which have rod-like structures. Thus, this study expands the diversity of possible silver nanocluster geometries that can be stabilized by multidentate DNA ligands.

## Experimental Methods

*Synthesis of Ag_N_-DNAs.* Ag_*N*_-DNAs were synthesized by mixing DNA oligomers (Integrated DNA Technologies) with AgNO_3_ in a 10 mM NH_4_OAc solution, followed by partially reducing the silver content with a 0.5 ratio of NaBH_4_. Final concentrations were 25 μM DNA, 100 μM AgNO_3_, and 50 μM NaBH_4_ for 800-Ag_*N*_-DNA and 35 μM DNA, 10 mM NH_4_OAc, 175 μM AgNO_3_, and 87.5 μM NaBH_4_ for 760-Ag_*N*_-DNA. Samples were stored in the dark at 4 °C until purification by high performance liquid chromatography (HPLC) (details in the SI) and then exchanged into 10 mM NH_4_OAc before further characterization.

*Optical Characterization.* Steady-state absorbance and emission spectra were recorded using a thermoelectrically cooled, fiber-coupled spectrometer (Ocean Optics QE65000). Absorbance spectra were collected using a DH-Mini (Ocean Insight) deuterium and tungsten halogen UV–vis–NIR light source. Fluorescence spectra were collected using a UV LED for universal UV excitation^21^ and an HPX-2000 xenon lamp (Ocean Insight) coupled with a Monoscan 2000 monochromator (Ocean Optics) for visible excitation. Time-resolved emission measurements were carried out with a FluoTime300 instrument from PicoQuant (see the SI for details). Circular dichroism (CD) measurements were performed on a Jasco J-810 CD spectrometer.

*Mass Spectrometry.* Electrospray ionization mass spectrometry (ESI-MS) was performed with a Waters Xevo G2-XS QTof. Samples were directly injected at 10 μL/min in negative ion mode with a 2 kV capillary voltage, 30 V cone voltage, and no collision energy. Spectra were collected from 1000 to 4000 *m*/*z* and integrated for 1 s. Source and desolvation temperatures were 80 and 150 °C, respectively. Gas flows were 45 L/h for the cone and 450 L/h for the desolvation. Samples were injected with 50 mM NH_4_OAc–MeOH (80:20) solution at pH 7 (for 760-Ag_*N*_-DNA, the solution also contained 0.1% HCOOH). Determination of the nanocluster size and charge was performed by fitting the calculated isotopic distribution of the Ag_*N*_-DNA to the experimental spectra (details in the SI). Calculated isotopic distributions were obtained from MassLynx using the chemical formula and corrected for the overall positive charge (oxidation state) of the complex.^2^

## Supporting Information Available

Materials and methods, HPLC chromatograms, additional steady-state and time-resolved photoluminescence spectra and decay fits, data for quantum yield determination, mass spectra, and calculated mass distributions (PDF)
