# Supplementary material for: DNA Stabilizes Eight-Electron Superatom Silver Nanoclusters with Broadband Downconversion and Microsecond-Lived Luminescence
Source: J Phys Chem Lett. 2022 Aug 29;13(35):8305–11. doi: 10.1021/acs.jpclett.2c02207 (PMC9465679; doi:10.1021/acs.jpclett.2c02207)
Supplement: Supplementary file 1 — jz2c02207_si_001.pdf [file jz2c02207_si_001.pdf]

Supporting Information: DNA Stabilizes 8-Electron Superatom Silver Nanoclusters with Broadband Downconversion and Microsecond-Lived Luminescence

Anna Gonzàlez-Rosell,[a] Rweetuparna Guha,[a] Cecilia Cerretani,[b] Vanessa Rück,[b] Mikkel B. Liisberg,[b] Benjamin B. Katz,[c] Tom Vosch,[b] Stacy M. Copp, [a,d,e]*

[a] *Department of Materials Science and Engineering, University of California, Irvine, CA 92697*

[b] *Nanoscience Center and Department of Chemistry, University of Copenhagen, Universitetsparken 5, 2100 Copenhagen, Denmark.*

[c] *Department of Chemistry, University of California, Irvine, CA 92697*

[d] *Department of Physics and Astronomy, University of California, Irvine, CA 92697*

[e] *Department of Chemical and Biomolecular Engineering, University of California, Irvine, CA 92697*

*Correspondence to stacy.copp@uci.edu

1. Experimental methods

1.1 Synthesis and Purification

Ag_N-DNAs are synthesized by mixing single-stranded DNA oligomers (Integrated DNA Technologies) with $AgNO_3$ (Sigma Aldrich) in a 10 mM ammonium acetate (NH_4OAc) solution (pH 7). After 15 minutes, a 0.5 molar fraction of $NaBH_4$ to $AgNO_3$ is added to partially reduce the silver cations. The ratio of the components in the final mixture is optimized for each sample to achieve maximal fluorescence brightness of the desired product. Samples were stored in the dark at 4 ºC for five days to maximize the chemical yield of the NIR product, followed by purification by high performance liquid chromatography (HPLC).

Purification of Ag_N-DNAs is essential to ensure that measured photophysical properties are not affected by the presence of synthesis byproducts, such as DNA oligomers, Ag^+-DNA complexes, Ag nanoparticles, and other fluorescent or nonfluorescent Ag_N-DNAs in solution. Purification is also critical to accurately determine the composition of the Ag_N-DNA of interest. Samples are purified a few days after synthesis (exact time depending on the Ag_N-DNA species).

Purification was performed on an Agilent 1260 Infinity system. We replaced the original fluorescence detector (FLD) for a Hamamatsu R13456 photomultiplier tube (PMT) in order to increase sensitivity > 600 nm, providing 250X signal enhancement at 750 nm. We used a Kinetex C18 column with 100 Å pore diameter, 5 µm particle size, and 50 mm × 46 µm dimensions (Phenomenex). The solvents used were MilliQ H_2O and MeOH, with 35 mM triethyl ammonium acetate (TEAA) as an ion-pairing agent in both. The *in-situ* absorbance spectra recorded by the diode-array detector and the fluorescence spectra confirmed that the fraction contains our NIR product of interest.

800-Ag_N-DNA:

The gradient was preceded by a 5 min pre-injection at 5% MeOH. Elution steps were the following: 0 – 2 min: 12% MeOH, 2 – 12 min: 12 – 27% MeOH, 12 – 14 min: 27 – 95 % MeOH, 14 – 19 min: 95% MeOH. The flow rate was 1.0 mL/min. The signals monitored for collection of the sample correspond to the characteristic UV and visible absorbance peaks and visible-NIR emission peaks: 260, 370, 410, and 460 nm for the UV/Vis diode array detector (DAD), and 780 and 530 nm for the fluorescence detector (FLD), exciting at 260 nm. The product was collected using the 780 nm emission signal, around 4.5 min, at 16% MeOH.

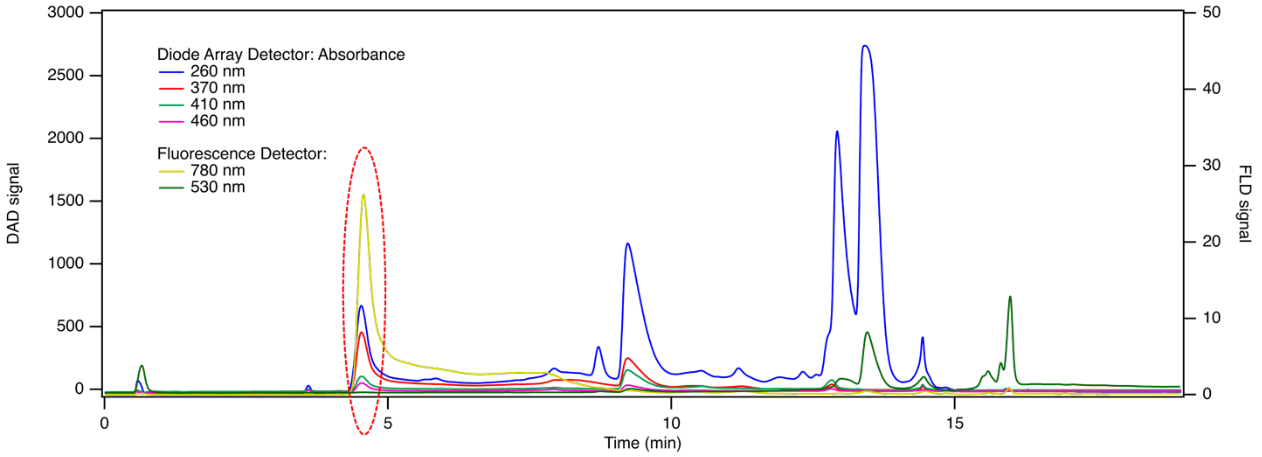

Figure S1: HPLC chromatograms of 800-Ag_NDNA. Dashed line shows the collected fraction. DAD signals correspond to absorbance through the DNA nucleobases (260 nm), and absorbance through the NIR nanocluster of interest (370, 410 and 460 nm). FLD signals correspond to the emission wavelengths of the NIR product of interest (780 nm) and a green-emitting product (530 nm).

760-Ag$_N$-DNA:

The sample was concentrated and purified 5 days after synthesis. 35 mM TEAA H_2O – MeOH gradient was used, with 5 min pre-injection at 5% MeOH. Time steps were the following: 0 – 2 min: 15% MeOH, 2 – 12 min: 15 – 30% MeOH, 12 – 14 min: 30 – 95% MeOH, 14 – 19 min: 95% MeOH, 19 – 24 min: 95 – 5% MeOH, and 3 min post-time at 5% MeOH. The flow rate was 1.0 mL/min. The signals were monitored at 260, 350, 410, and 460 nm for the DAD and 780 and 530 nm for the FLD (λ_{exc} = 260 nm). The product was collected using the 780 nm emission signal, around 2.7 min, at 16% MeOH.

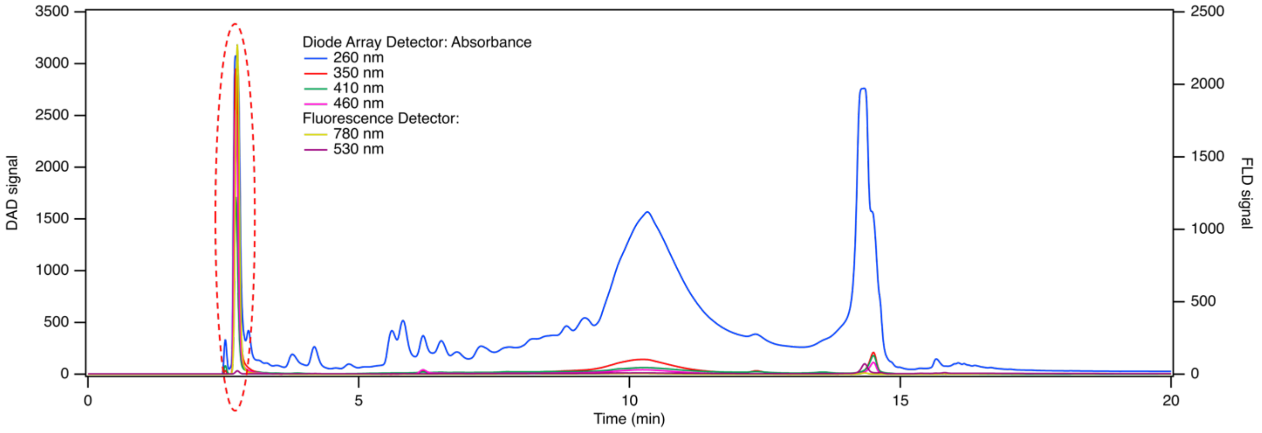

Figure S2: HPLC chromatograms of 760-Ag$_N$-DNA. Dashed line shows the collected fraction. DAD signals correspond to absorbance through the DNA nucelobases (260 nm), and absorbance through the NIR nanocluster of interest (350, 410 and 460 nm). FLD signals correspond to the emission wavelengths of the NIR product of interest (780 nm) and a green emissive side product (530 nm).

After purification, samples were solvent exchanged into 10 mM NH_4OAc using 3 kDa spin filters (Amicon, Millipore Sigma). The fact that absorption and emission spectra in Figure S3 overlap reasonably well confirms that both purifications were successful.

1.2 Spectroscopic measurements

All measurements for both 800-Ag$_N$-DNA and 760-Ag$_N$-DNA were performed in 10 mM ammonium acetate (NH_4OAc) aqueous solution at 25 ^oC or room temperature.

Steady-state absorbance and emission spectra (**Figures 1 and 2**) were recorded using a thermoelectrically cooled, fiber-coupled spectrometer (Ocean Optics QE65000), in a quartz cuvette with 1 mm optical path length (Starna Cells). Absorbance spectra (**Figure 2**) were collected using a DH-Mini (Ocean Insight) deuterium & tungsten halogen UV-Vis-NIR light source. Note

that the absorbance signal below 250 nm might be slightly distorted due to higher attenuation of the fiber optic cable at these wavelengths. Emission spectra (**Figure 1**) were collected using a UV LED for universal UV-excitation[1] and an HPX-2000 xenon lamp (Ocean Insight) coupled with a Monoscan 2000 monochromator (Ocean Optics) for visible excitation.

Time-resolved emission measurements were carried out with a FluoTime300 instrument (PicoQuant), using picosecond-pulsed diode lasers in both time-correlated single photon counting (TCSPC) mode (Figure S5) and burst mode approach (Figures 4, S7 and S8), and a Xenon flash lamp (Figure S6) as excitation source.

Circular dichroism (CD) measurements were performed on a Jasco J-810 CD spectrometer. CD spectra were recorded from 190 to 800 nm at 20 °C in a cuvette with 1 mm optical path length (Starna Cells) with a scanning rate of 100 nm/min. The CD spectrum of each Ag_N-DNA is the average of three scans and a manual baseline correction (contribution from 10 mM NH_4OAc) was applied.

Absorption spectra in Figure S3 were measured either with a Cary300 UV-Vis spectrophotometer from Agilent Technologies or a Lambda1050 instrument from Perkin Elmer, using a deuterium lamp for ultraviolet radiation and a tungsten-halogen lamp for visible and near-infrared (NIR) radiation. Excitation spectra in Figure S3 were measured with a QuantaMaster400 instrument from PTI/Horiba with a Xenon arc lamp and were corrected for the wavelength dependency of the detector system. Steady-state emission measurements of 800-Ag_N-DNA in Figure S4 were performed in 10 mM NH_4OAc at 25 ºC, using a FluoTime300 instrument from PicoQuant, exciting with a Xenon arc lamp at 375, 405, 470 and 510 nm. A long-pass 514 nm filter (Semrock, LP02-514RU-25) was placed in the emission path to remove the second harmonic of the lamp. Finally, the spectra were corrected for the wavelength dependency of the detector.

1.2.1 Time-resolved emission measurements:

Burst mode measurements:

800-Ag_N-DNA: The luminescence trace in Figure 4A was measured at λ_{em} = 810 nm, exciting at 374.3 nm (PicoQuant, LDH-P-C-375). A long-pass 514 nm filter (Semrock, LP02-514RU-25) was used in the emission path. The repetition rate of the laser was set to 20 MHz, with an effective

sync rate of 10 kHz. The laser burst lasted 25 μs and consisted of 500 pulses (*i.e.,* every pulse was separated by 50 ns) followed by 75 μs where the laser was switched off (25 % duty cycle). The ratio between the number of pulses and the repetition rate of the excitation laser defines the burst length, while the effective sync rate determines the overall length of the measurements (100 μs, in this case). The integration time was chosen to be 300 s. The intensity decay upon turning off the laser (at 25 μs) was tail-fitted with a mono-exponential model.

760-Ag$_N$-DNA: Figure 4B shows the emission intensity acquired at 760 nm in burst mode, exciting at 374.3 nm (PicoQuant, LDH-P-C-375). A long-pass 633 nm filter (Semrock, LP02-633RU-25) was used in the emission path. The repetition rate of the laser was chosen to be 40 MHz, with an effective sync rate of 1 kHz. The laser burst lasted 400 μs and comprised 16,000 pulses (*i.e.,* every pulse was separated by 25 ns) followed by 600 μs where the laser was switched off (40 % duty cycle). The integration time was chosen to be 900 s. The luminescence decay upon turning off the laser (at 400 μs) was tail-fitted mono-exponentially.

TCSPC with ps-pulsed diode lasers and Xenon flash lamp measurements:

800-Ag$_N$-DNA: Luminescence decay curves reported in Figure S12 were measured in TCSPC mode. The decay traces were monitored at λ_{em}=800 nm, exciting at four different wavelengths: 374.3 nm (PicoQuant, LDH-P-C-375), 407.2 nm (PicoQuant, LDH-P-C-405), 467.2 nm (PicoQuant, LDH-P-C-470) and 507.5 nm (PicoQuant, LDH-P-C-510). The repetition rate of the lasers was set to 30 kHz and the integration time was chosen to be 600 s. Additionally, a long-pass 532 nm filter (Semrock, BLP01-532R-25) was introduced in the emission path. All decays were tail-fitted mono-exponentially with FluoFit v.4.6 software from PicoQuant, resulting in decay time values of 2.35-2.36 μs.

760-Ag$_N$-DNA: The microsecond decay shown in Figure S13 was measured at λ_{em}=760 nm, exciting at 355 nm with a Xenon flash lamp (repetition rate of 300 Hz) and integrated for 900 s. Two filters were used: a long-pass 325 nm (Semrock, BLP01-325R-25) in the excitation path and a long-pass 633 nm filter (Semrock, LP02-633RU-25) in the emission path. The decay was tail-fitted mono-exponentially using the above-mentioned software. The decay time,[2] τ, was determined to be 18.7 ± 0.1 μs.

O_2 removal experiment for 800-Ag$_N$-DNA was performed to shed light on the origin of the long-lived luminescence. The cuvette filled with 800-Ag$_N$-DNA in 10 mM NH_4OAc was connected to

a water jet pump that lowered the head space pressure in the cuvette and hence decreased the amount of dissolved O_2 in solution as a function of time. Burst mode and TCSPC decays were measured at 25 ^oC before removing O_2 and after 10, 20 and 30 min of pumping. All data are reported in Figure S16.

The luminescence traces in Figure S16A were measured at λ_{em} = 810 nm, exciting at 374.3 nm (PicoQuant, LDH-P-C-375). A long-pass 514 nm filter (Semrock, LP02-514RU-25) was used in the emission path. The repetition rate of the laser was set to 20 MHz, with an effective sync rate of 10 kHz. The laser burst comprised 500 pulses in 25 µs, then the laser was switched off for 75 µs (25 % duty cycle). The integration time was set to 300 s. All intensity decays upon turning off the laser (at 25 µs) were tail-fitted with a mono-exponential model, using FluoFit v.4.6 software from PicoQuant. The decay times (Table S2) were found to be constant as a function of exposure time to the low pressure generated by the pump.

Intensity decays (Figure S16B) were also measured at 810 nm in TCPSC mode, before (0 min) and after molecular oxygen removal (10, 20 and 30 min). The sample was excited with a ps-pulsed laser at 374.3 nm with a repetition rate of 100 kHz. Every decay was integrated for 600 s, and a long-pass 514 nm filter (Semrock, LP02-514RU-25) was added in the emission path. The decays were tail-fitted mono-exponentially with Fluofit v.4.6 software (PicoQuant). The decay times, reported in Table S1, are consistent with the values obtained with the burst mode approach.

1.2.2 Quantum Yield (QY) measurements and calculations

Luminescence quantum yield (QY) values for 800-Ag_N-DNA and 760-Ag_N-DNA were determined at room temperature in 10 mM NH_4OAc, using 736-Ag_N-DNA in the same medium as reference (QY_{ref}= 0.26)[3]. Given the large energy difference between the main absorption peak of the Ag_N-DNAs of interest and the emission band, it was not possible to use any standard reference dye.[4] Thus, 736-Ag_{16}-DNA was chosen as the reference compound due to its considerable Stokes shift, absorption peaks in the same wavelength region as 800-Ag_N-DNA and 760-Ag_N-DNA, and similar emission range. The emission spectra were measured exciting at 374.3 nm, whereas the quantum yield of the reference was previously calculated at λ_{exc}= 532 nm.[3] For this reason, the value of QY_{ref} was corrected for the difference between the excitation and absorption efficiency at 374.3 nm (see

below). Note that due to the large difference in excitation and emission wavelength and the lack of good references, the reported values should be considered as approximate values.

Absorption spectra were acquired with a Cary300 UV-Vis spectrophotometer, excitation spectra were carried out with a QuantaMaster400 instrument from PTI/Horiba, while emission measurements were performed on the home-built confocal microscope to overcome the low detector efficiency of the FluoTime300 instrument (PicoQuant) above 800 nm. A brief description of the home-built setup is given below.

The output of a 374.3 nm picosecond-pulsed laser diode (LDH-P-C-375, PicoQuant) was expanded (BE05M-A, Thorlabs), cleaned up by a 330-385 nm band pass filter and directed towards the microscope (IX71, Olympus). The beam was reflected by a 30:70 beam splitter (Omega Optical, XF122) and sent through an air objective (Olympus, CPlanFLN 10x, NA= 0.3). A standard 1 cm fluorescence quartz cuvette (Hellma) filled with solutions of 736-Ag_{16}-DNA, 800-Ag_N-DNA or 760-Ag_N-DNA was placed on top of the microscope sample stage, and the laser was focused 1 mm into the solutions ensuring that the spectra of the three samples were recorded under identical conditions. The objective that focused the laser into the samples and reference solutions was the same that collected the luminescence. The laser light was blocked by a 435 nm long-pass filter, and out-of-focus light was blocked by a 100 μm pinhole. The luminescence was sent through a spectrograph (SP 2356 spectrometer, 300 grooves/mm, Acton Research) onto a nitrogen cooled CCD camera (SPEC-10:100B/LN-eXcelon, Princeton Instruments) to record the spectra. Finally, the emission spectra were intensity corrected as reported previously.[5]

The luminescence quantum yield of 800-Ag_N-DNA and 760-Ag_N-DNA was calculated using the following formula[4]:

$$\boldsymbol{QY} = \frac{\boldsymbol{F}}{\boldsymbol{f_A}} \cdot \frac{\boldsymbol{f_{A,ref}}}{\boldsymbol{F_{ref}}} \cdot \frac{\boldsymbol{n^2}}{\boldsymbol{n^2_{ref}}} \cdot \boldsymbol{QY_{ref}} \cdot \left(\frac{\boldsymbol{exc_{\lambda exc}}}{\boldsymbol{abs_{\lambda exc}}}\right)$$

where $\boldsymbol{QY}$ represents the quantum yield, $\boldsymbol{F}$ is the integrated emission spectrum (*i.e.,* the area under the spectrum), $\boldsymbol{f_A}$ defines the fraction of absorbed light at the excitation wavelength (λ_{exc}≈374 nm), and $\boldsymbol{n}$ is the refractive index of the medium. Quantities with or without the ***ref*** subscript indicate the reference compound or the sample, respectively. $\left(\frac{\boldsymbol{exc_{\lambda exc}}}{\boldsymbol{abs_{\lambda exc}}}\right)$ represents the ratio between the

excitation and absorption values (both normalized to 1 at 525 nm) of the reference nanocluster at the excitation wavelength.

Only one point for the integrated emission counts and one point for the fraction of absorbed light were used in the quantum yield calculations for both Ag_N-DNAs. A luminescence QY of 1% was found for 800-Ag_N-DNA, whereas 4% was the value determined for 760-Ag_N-DNA. See Figures S5 and S6 for the actual data.

2. Results and discussion

2.1 Steady-state measurements

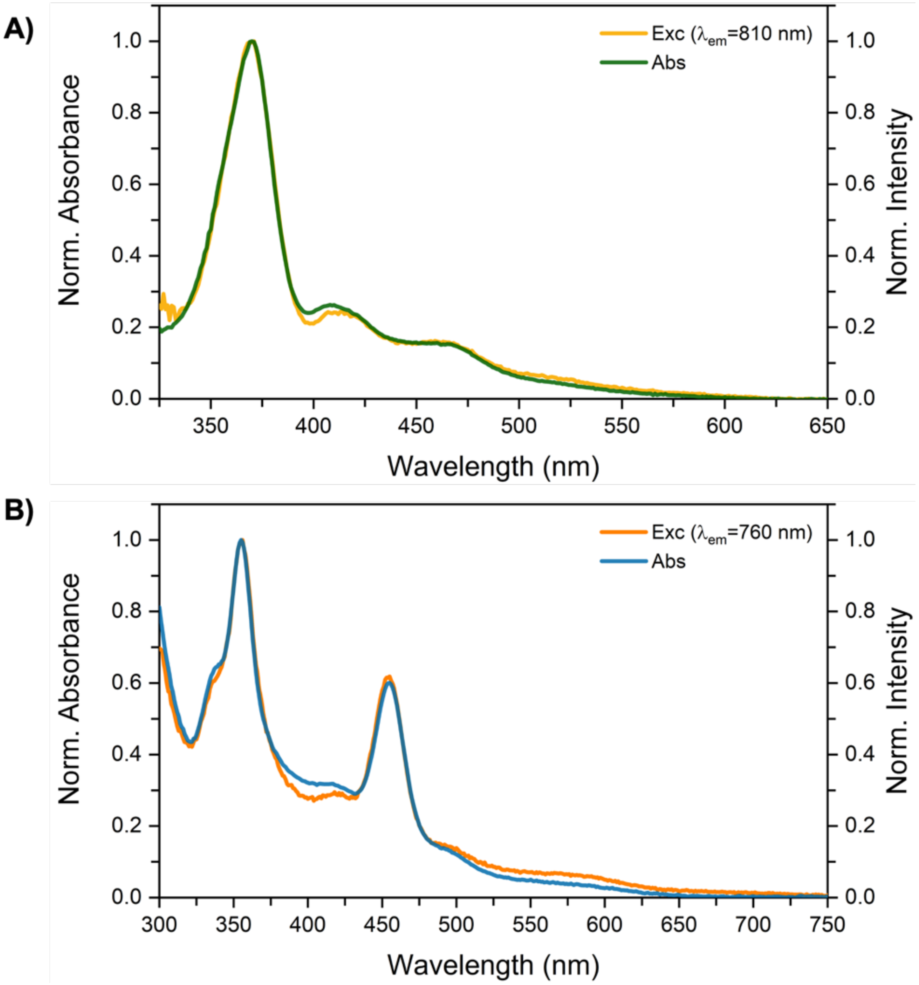

Figure S3: Comparison of excitation and absorbance spectra to ensure that Ag_N-DNAs are compositionally pure. **A)** Overlay of normalized absorption (green) and excitation (yellow) spectra (λ_{em}=810 nm) of 800-Ag_N-DNA in 10 mM NH_4OAc at room temperature. Both spectra are normalized for the maximum at 370 nm. **B)** Overlay of normalized absorption (blue) and excitation (orange) spectra (λ_{em}=760 nm) of 760-Ag_N-DNA in 10 mM NH_4OAc at room temperature. Both spectra are normalized for the maximum at 355 nm.

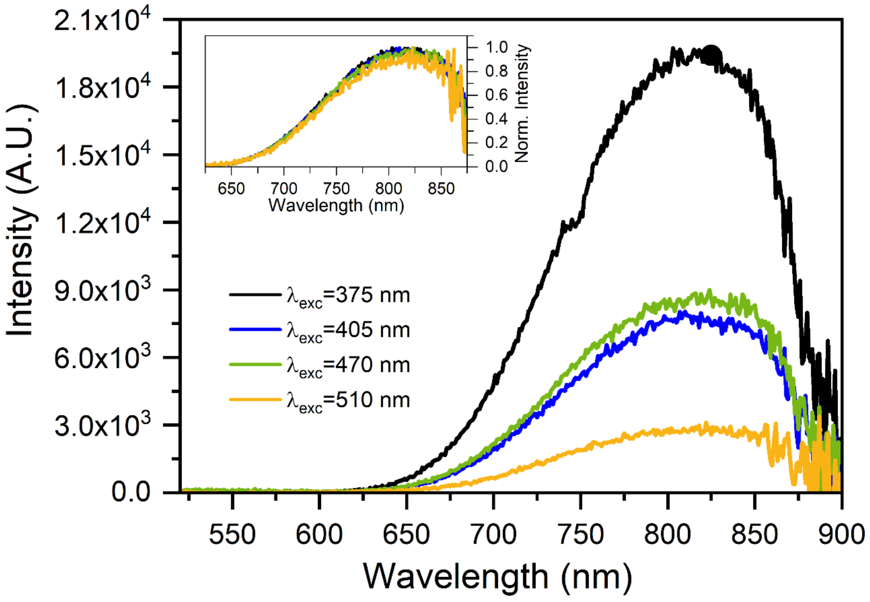

Figure S4: Emission spectra of 800-Ag$_N$-DNA in 10 mM NH_4OAc at room temperature, exciting at different wavelengths with a Xenon arc lamp: 375, 405, 470 and 510 nm. A long-pass 514 nm filter (Semrock, LP02-514RU-25) was added in the emission path. The insert shows the same spectra, but with the emission maxima normalized to 1.

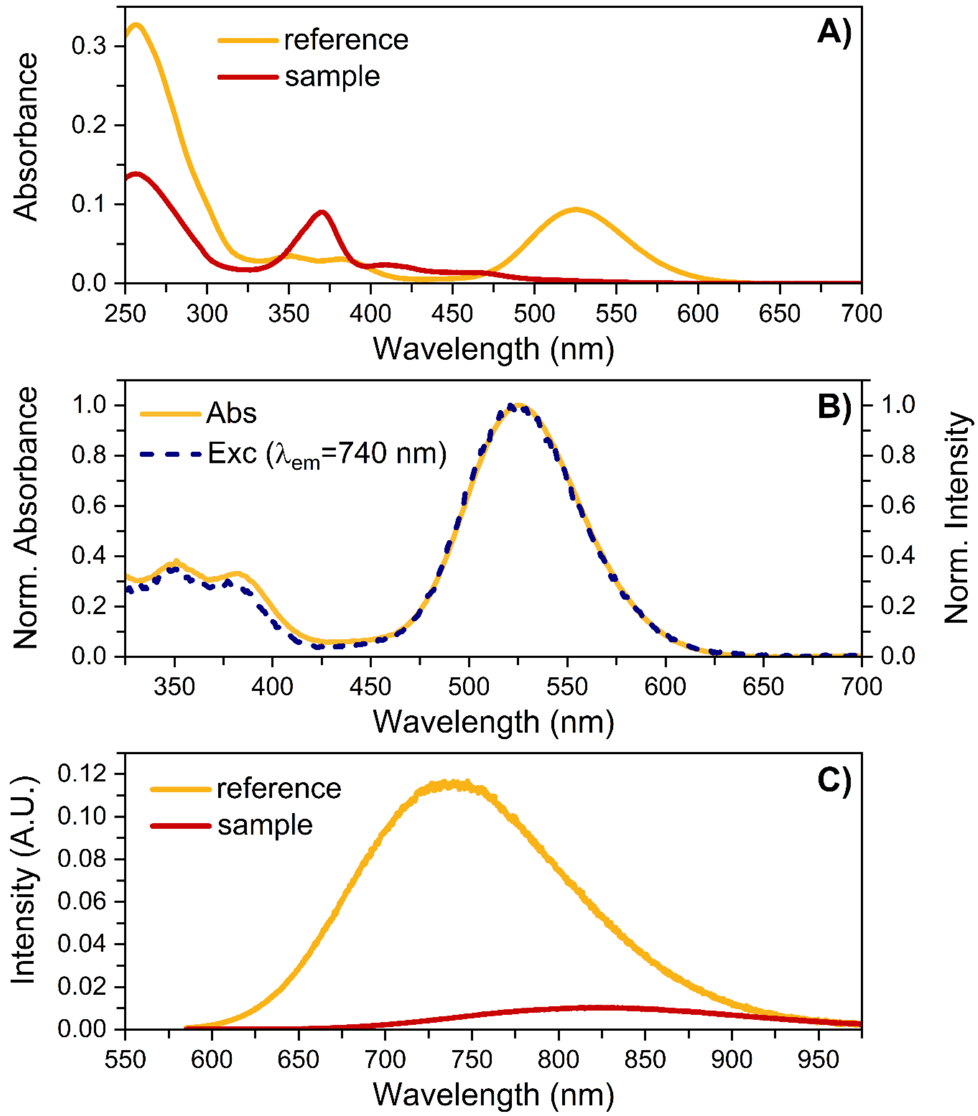

Figure S5: Luminescence quantum yield measurements of 800-Ag$_N$-DNA in 10 mM NH_4OAc at 25 ºC, using 736-Ag$_{16}$-DNA in the same medium as reference compound (QY_{ref}=0.26)[1]. **A)** Absorption spectra of 800-Ag$_N$-DNA (red) and 736-Ag$_{16}$-DNA (yellow). **B)** Overlay of absorption (solid yellow) and excitation (dashed navy) spectra of 736-Ag$_{16}$-DNA, normalized for the maximum at 525 nm. The excitation spectrum was monitored at λ_{em}=740 nm. **C)** Emission spectra of 800-Ag$_N$-DNA (red) and 736-Ag$_{16}$-DNA (yellow) measured on a single-molecule sensitive microscope, exciting at 374.3 nm with a picosecond-pulsed laser (PicoQuant).

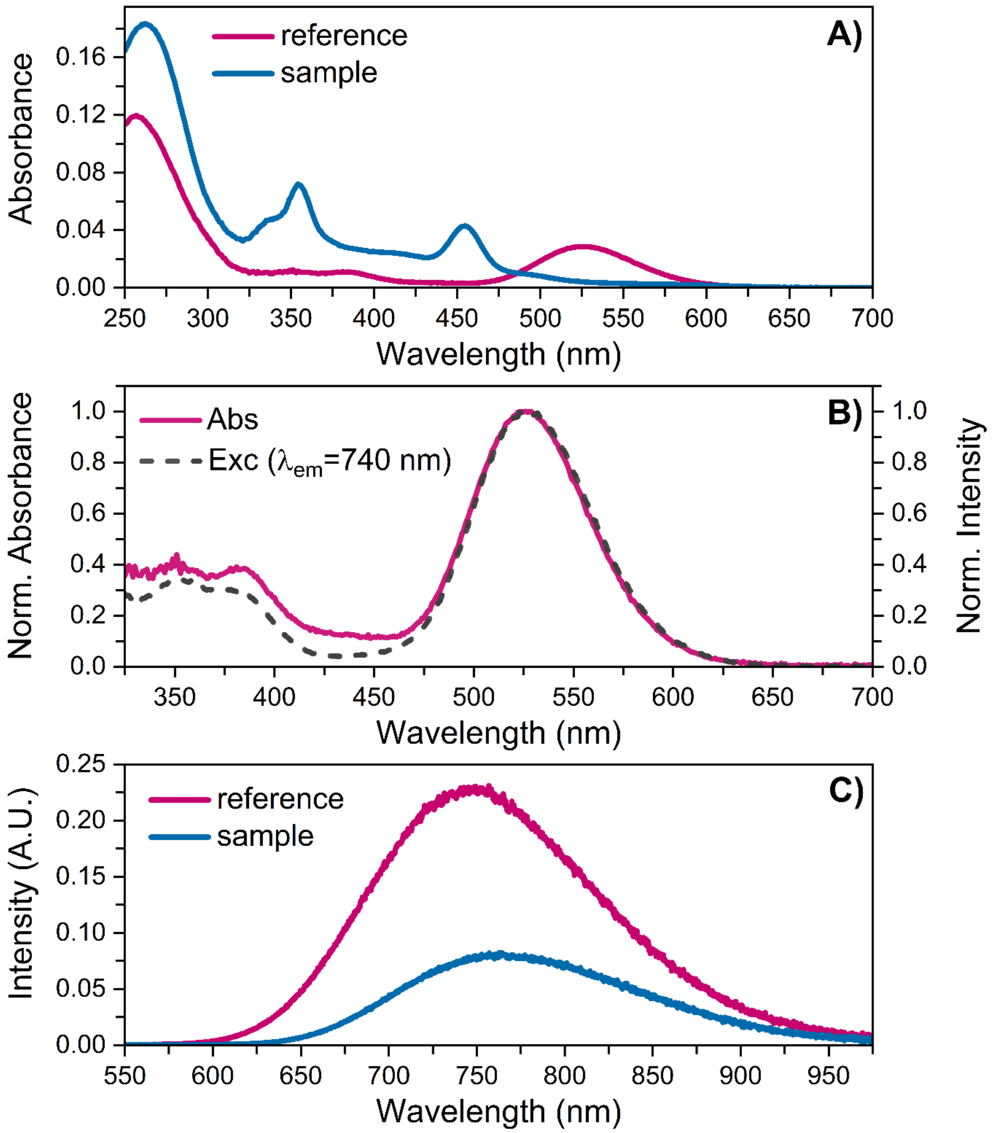

Figure S6: Luminescence quantum yield measurements of 760-Ag$_{N}$-DNA in 10 mM NH_4OAc at 25 ºC, using 736-Ag$_{16}$-DNA in the same medium as reference compound (QY_{ref}=0.26)[1]. **A)** Absorption spectra of 760-Ag$_{N}$-DNA (blue) and 736-Ag$_{16}$-DNA (purple). **B)** Overlay of absorption (solid purple) and excitation (dashed gray) spectra of 736-Ag$_{16}$-DNA, normalized for the maximum at 525 nm. The excitation spectrum was monitored at λ_{em}=740 nm. **C)** Emission spectra of 760-Ag$_{N}$-DNA (blue) and 736-Ag$_{16}$-DNA (purple) measured on a single-molecule sensitive microscope, exciting at 374.3 nm with a picosecond-pulsed laser (PicoQuant).

2.2 Mass Spectrometry

Determination of Ag_N-DNA composition and charge was performed by fitting the calculated isotopic distribution of the Ag_N-DNA to the experimental spectra. Calculated isotopic distributions were obtained from MassLynx using the chemical formula and corrected for the overall positive charge (oxidation state) of the complex.[6] All peaks in the mass spectra (below) were identified as dimers ($n_s = 2$) with different numbers of total silvers (N).

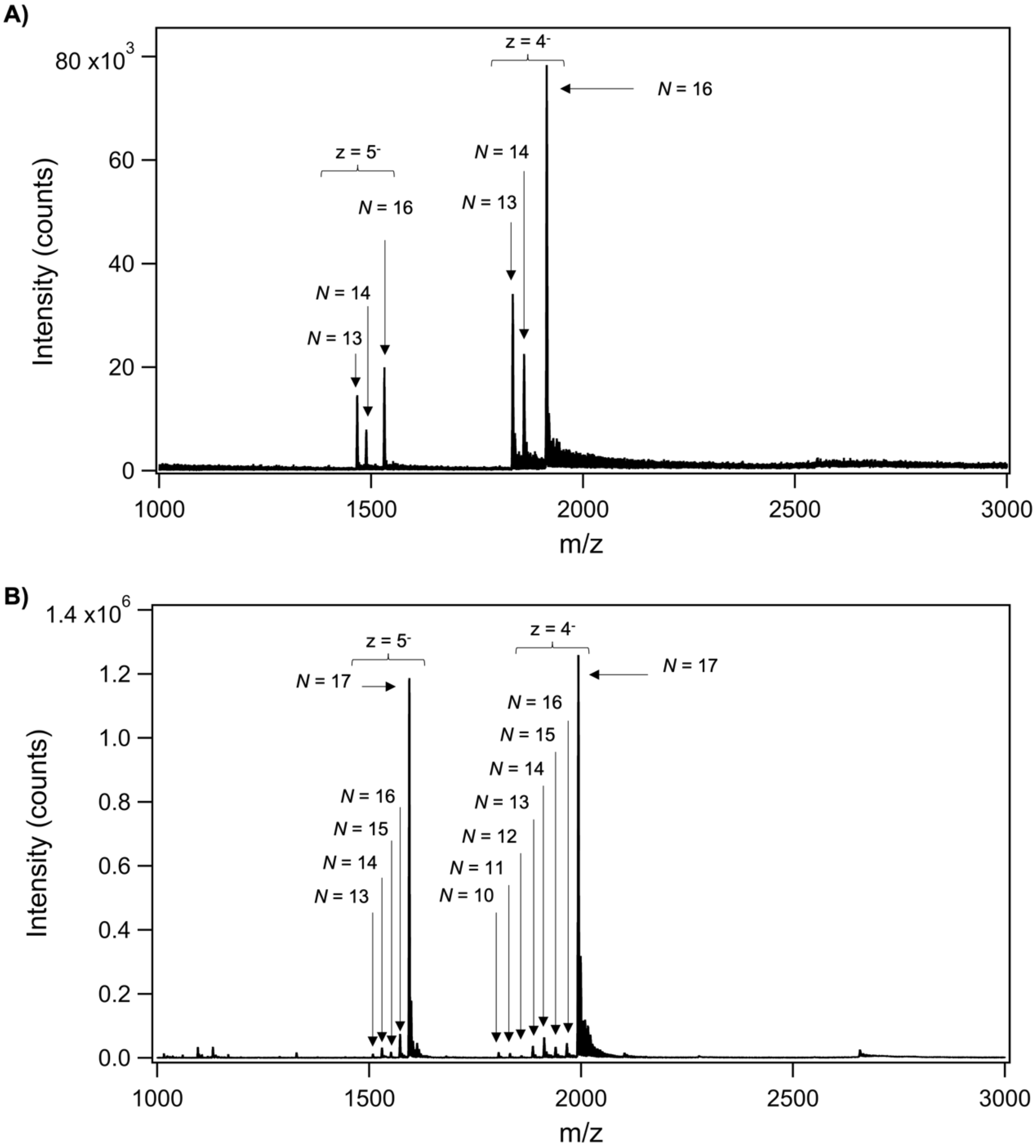

Figure S7: Mass spectra of **A)** 800-Ag_N-DNA and **B)** 760-Ag_N-DNA. All peaks correspond to dimers with various numbers of silvers, *N*.

To determine the overall charge of the nanocluster (Q_{cl}), we fitted single Gaussians to the experimental mass spectral peaks and compared the centers of these Gaussian fits to the centers of Gaussian fits of isotopic distributions calculated for a range of Q_{cl} values; this procedure was previously introduced to determine Q_{cl} for Ag_N-DNAs.[7] Table S1 provides fitting parameters for the dominant experimental mass spectral peaks as well as fitting parameters for calculated distributions for $Q_{cl} = 0$, the best fit Q_{cl}, and ± 1 proton of the best fit. For both species at $z = 5^-$ and $z = 4^-$ charge states, the closest match to experimental data corresponds to products with $N_0 =$ 8 effective neutral silver atoms.

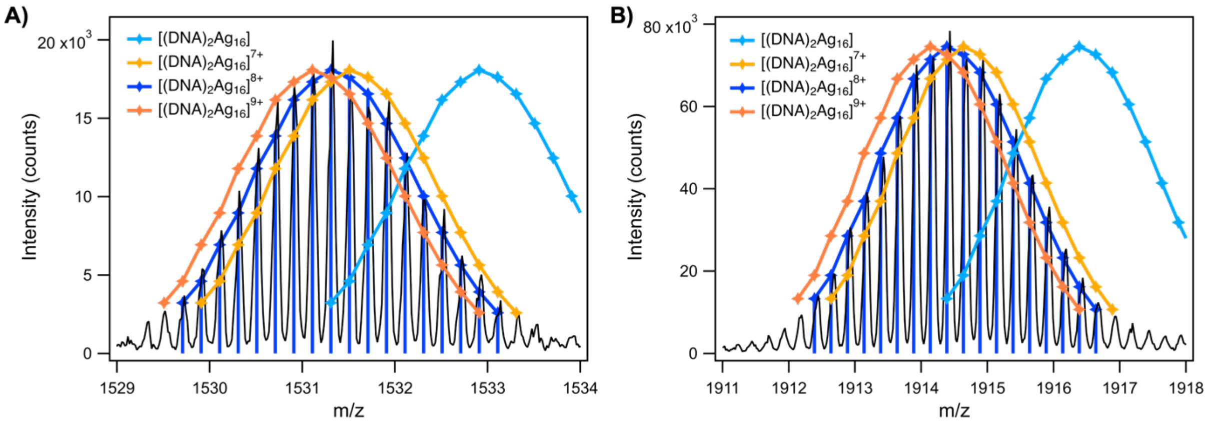

Figure S8: Experimental isotopic distribution (black curve) of 800-Ag_N-DNA at **A)** $z = 5^-$ and **B)** $z = 4^-$. Colored curves show calculated isotopic distributions from MassLynx using the chemical formula $C_{192}H_{246}N_{72}O_{114}P_{18}Ag_{16}$ with overall nanocluster charges of 0 (light blue), 7^+ (light orange), 8^+ (dark blue), and 9^+ (dark orange). A total charge of 8^+ provides the best fit to the data, as can be seen by comparing experimental and calculated values in Table S1.

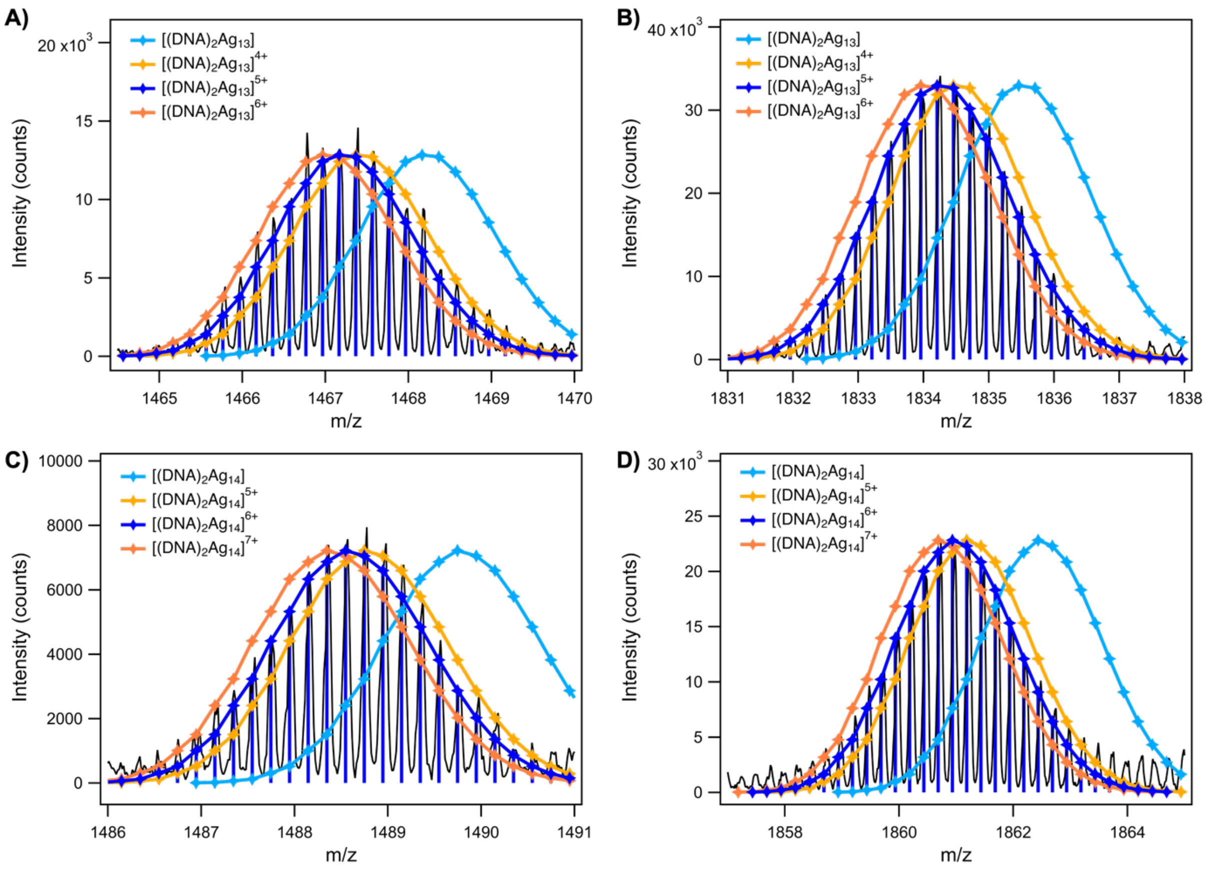

Figure S9: Experimental isotopic distributions (black curves) for minor peaks of 800-Ag$_N$-DNA mass spectra, corresponding to a dimer with N = 13 at **A**) z = 5$^-$ and **B**) z = 4$^-$, and a dimer with N = 14 at **C**) z = 5$^-$ and **D**) z = 4$^-$. Dark blue lines correspond to the calculated isotopic distribution using MassLynx, corrected for the total charge of the nanocluster. Colored curves show calculated isotopic distributions with different overall nanocluster charges.

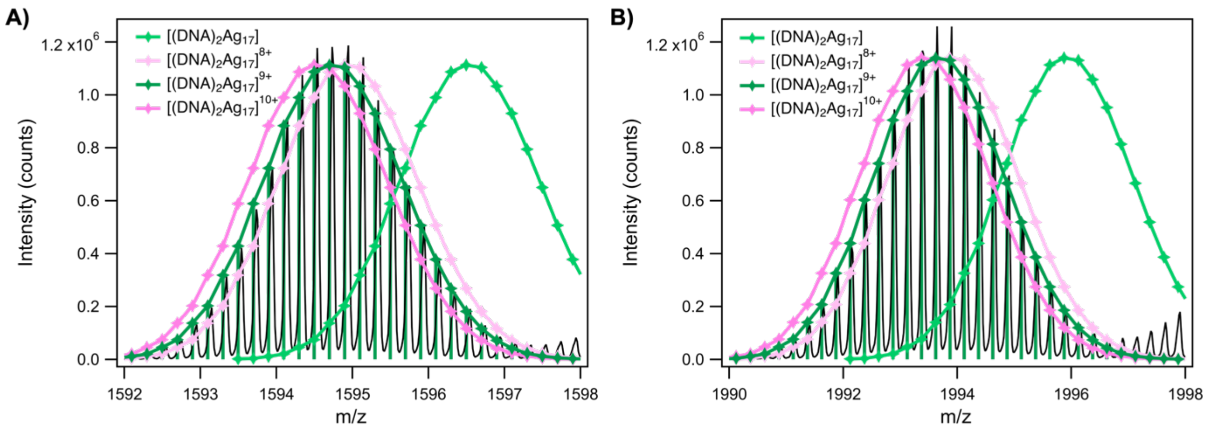

Figure S10: Experimental isotopic distribution (black curve) of 760-Ag$_N$-DNA at **A)** $z = 5^-$ and **B)** $z = 4^-$. Colored curves show calculated isotopic distributions from MassLynx using the chemical formula $C_{196}H_{244}N_{86}O_{112}P_{18}Ag_{17}$ with overall nanocluster charges of 0 (light green), 8+ (light pink), 9+ (dark green), and 10+ (dark pink). A total charge of 9+ provides the best fit to the data. Experimental and calculated values in Table S1.

Table S1: Center of Gaussian fits, x_0, to the dominant peaks in the experimentally measured mass spectra of 800-Ag$_N$-DNA (Figure S8) and 760-Ag$_N$-DNA (Figure S10), and to the calculated mass distributions for different overall nanocluster charges, Q_{cl}. Rightmost column compares x_0 for the experimental mass spectral peak to x_0 for the calculated mass distribution at the specific Q_{cl}. Bold text corresponds to experimental values and the corresponding best matching Q_{cl}. Note that $N_0 = N - Q_{cl}$

Ag$_N$-DNA	Charge state	Fit	x_0	(Experimental x_0) – (Calculated x_0)
800-Ag$_N$-DNA	$z = 5^-$	**Experimental**	**1531.337 ± 0.007**	
		$Q_{cl} = 0$	1532.946 ± 0.003	-1.609
		$Q_{cl} = 7$	1531.546 ± 0.003	0.209
		**$Q_{cl} = 8$**	**1531.346 ± 0.003**	**-0.009**
		$Q_{cl} = 9$	1531.146 ± 0.003	0.191
	$z = 4^-$	**Experimental**	**1914.50 ± 0.07**	
		$Q_{cl} = 0$	1916.435 ± 0.004	-1.935
		$Q_{cl} = 7$	1914.685 ± 0.004	0.185
		**$Q_{cl} = 8$**	**1914.435 ± 0.004**	**0.065**
		$Q_{cl} = 9$	1914.185 ± 0.004	0.315
760-Ag$_N$-DNA	$z = 5^-$	**Experimental**	**1594.79 ± 0.08**	
		$Q_{cl} = 0$	1596.547 ± 0.003	-1.757
		$Q_{cl} = 8$	1594.947 ± 0.003	0.157
		**$Q_{cl} = 9$**	**1594.747 ± 0.003**	**0.043**
		$Q_{cl} = 10$	1594.547 ± 0.003	0.243
	$z = 4^-$	**Experimental**	**1993.7 ± 0.1**	
		$Q_{cl} = 0$	1995.935 ± 0.003	-2.235
		$Q_{cl} = 8$	1993.935 ± 0.003	0.235
		**$Q_{cl} = 9$**	**1993.685 ± 0.003**	**0.015**
		$Q_{cl} = 10$	1993.435 ± 0.003	0.265

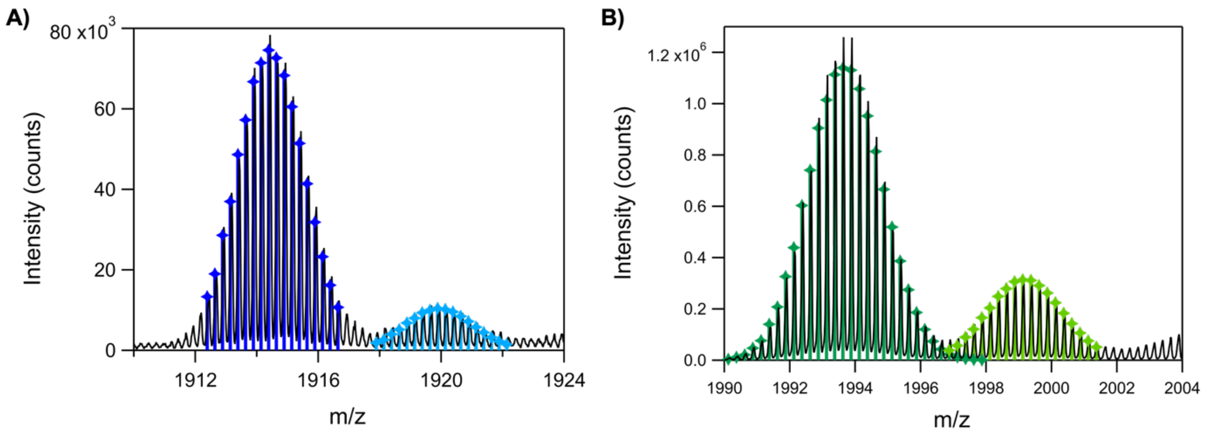

Figure S11: Ag_N-DNA products are well-separated from higher-mass salt adducts. Experimental isotopic distributions (black curve) of the main product and Na^+ adduct for **A)** 800-Ag_N-DNA and **B)** 760-Ag_N-DNA at $z = 4^-$. Dark blue (A) and dark green (B) lines correspond to the calculated isotopic distributions for the main Ag_N-DNA products using MassLynx, corrected for the total charge of the nanoclusters. Light blue (A) and light green (B) lines correspond to the calculated isotopic distribution for Ag_N-DNAs with one Na^+ cation, corrected for the total charge of the nanoclusters.

2.3 Time-resolved measurements

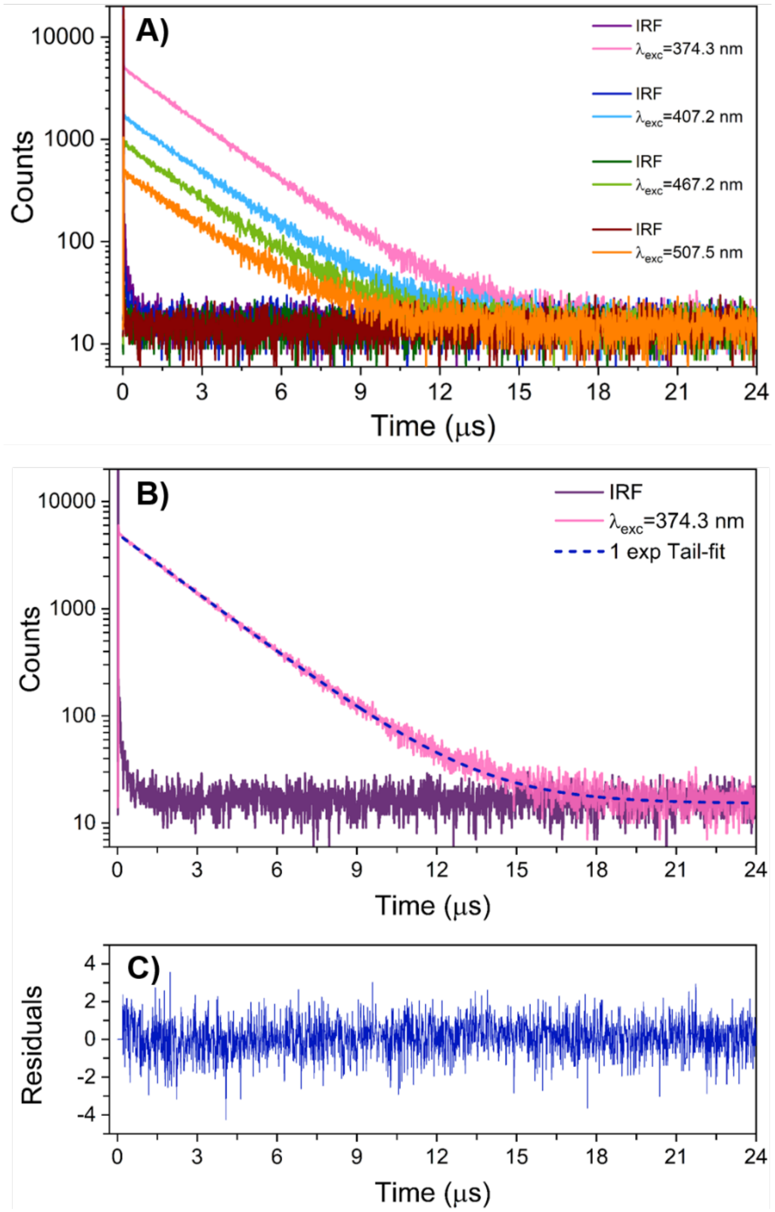

Figure S12: Time-resolved data of 800-Ag_N-DNA in 10 mM NH_4OAc at 25 ºC. **A)** Luminescence decay curves monitored at λ_{em}=800 nm, exciting at four different wavelengths: 374.3 nm (PicoQuant, LDH-P-C-375), 407.2 nm (PicoQuant, LDH-P-C-405), 467.2 nm (PicoQuant, LDH-P-C-470) and 507.5 nm (PicoQuant, LDH-P-C-510). The repetition rate of the lasers was set to 30 kHz and the integration time was chosen to be 600 s. Additionally, a long-pass 532 nm filter (Semrock, BLP01-532R-25) was introduced in the emission path. All decays were tail-fitted mono-exponentially with FluoFit v.4.6 software from PicoQuant, resulting in decay time values of 2.35-2.36 μs (See Table S2). **B)** Decay, exciting at 374.3 nm, and relative IRF, along with the mono-exponential tail-fit function (dashed blue). The residuals of the fit are reported in **C).**

Table S2: Values related to mono-exponential tail-fits of luminescence decays for 800-Ag$_N$-DNA in 10 mM NH_4OAc. The decay curves were recorded at λ_{em}=800 nm at T=25 ºC, exciting at four different wavelengths with picosecond-pulsed lasers.

λ_{exc} (nm)	A_1 (Counts)	τ_1 (µs)	χ^2
**374.3**	4565.6 ± 38.0	2.351 ± 0.015	0.981
**407.2**	1525.7 ± 18.3	2.363 ± 0.023	0.948
**467.2**	865.8 ± 17.0	2.349 ± 0.038	0.976
**507.5**	444.9 ± 12.6	2.363 ± 0.058	0.994

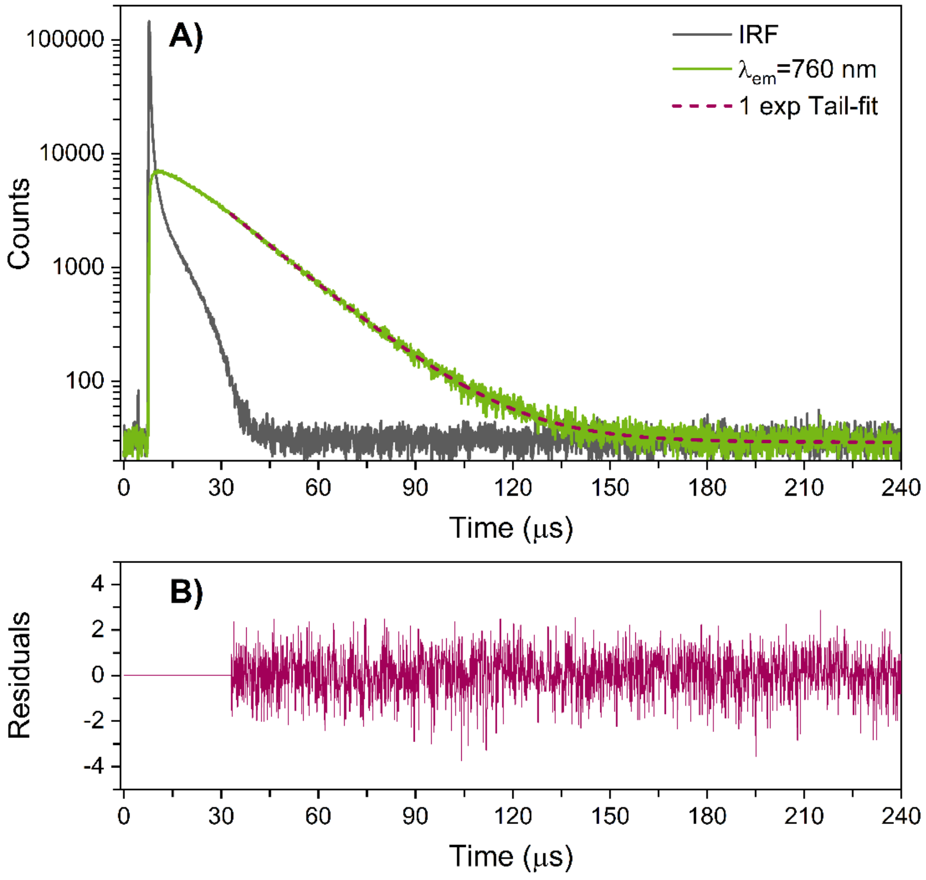

Figure S13: 760-Ag$_N$-DNA in 10 mM NH_4OAc at 25 ºC. **A)** Luminescence decay curve recorded at 760 nm, exciting at 355 nm with a Xenon flash lamp (repetition rate=300 Hz). The decay (light green) and IRF (dark gray) were integrated for 900 s. Two filters were used: a long-pass 325 nm (Semrock, BLP01-325R-25) in the excitation path and a long-pass 633 nm filter (Semrock, LP02-633RU-25) in the emission path. The decay was tail-fitted with a mono-exponential function (pink dashed line), and the related residuals (pink) are shown in **B)**. The decay time,[2] τ, was found to be 18.7 µs (A_1=2914.0 ± 27.9 counts; τ_{1}=18.710 ± 0.142 µs; χ^2=0.921).

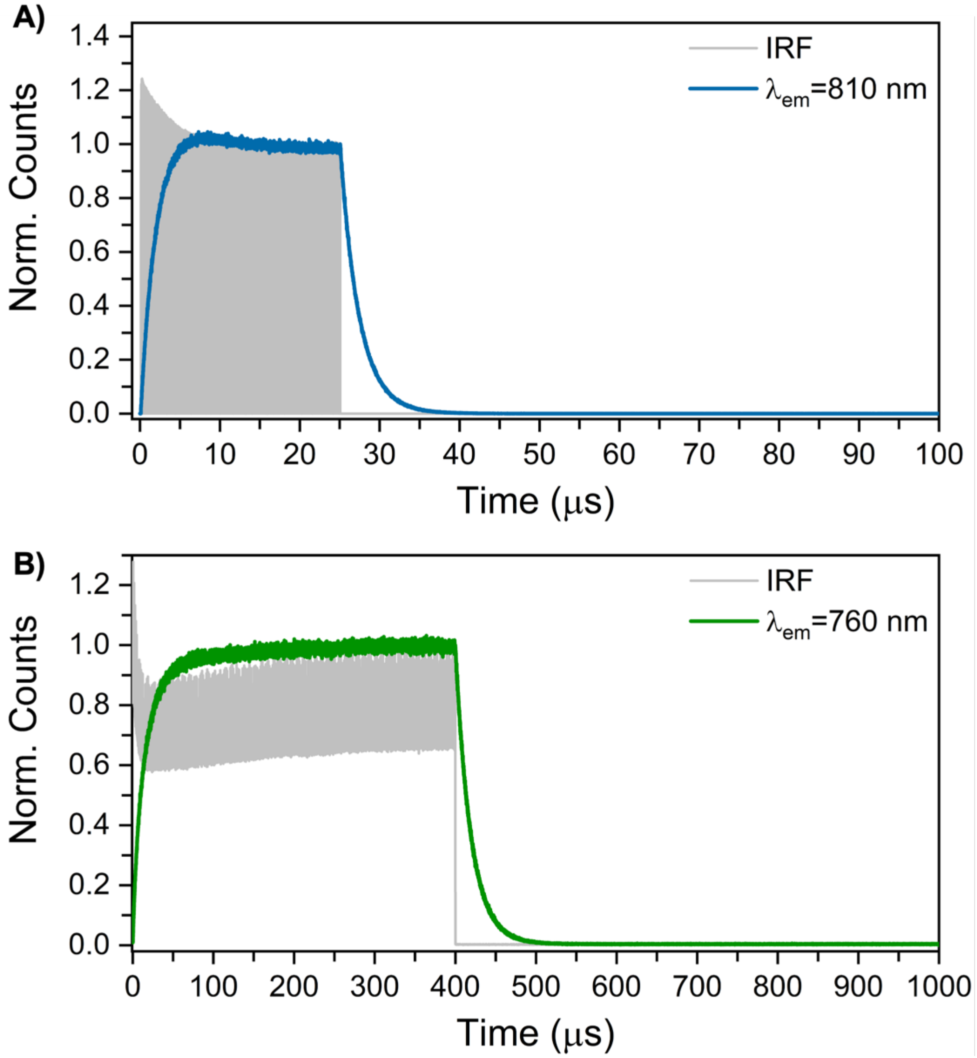

Figure S14: Decay curves of **A)** 800-Ag$_N$-DNA and **B)** 760-Ag$_N$-DNA measured in burst mode, exciting at 374.3 nm with a picosecond-pulsed laser (PicoQuant). Measurements were performed at 25 ºC in 10 mM NH4OAc. These are the same data reported in Figure 4, along with the corresponding IRFs (gray) and the full-time ranges. Note that the IRFs are not ideally flat for this laser, but this is not a problem since the decays were only tail-fitted from the point the laser burst ends. The time it takes to establish the steady-state equilibrium (rise) cannot be used in this case. [8]

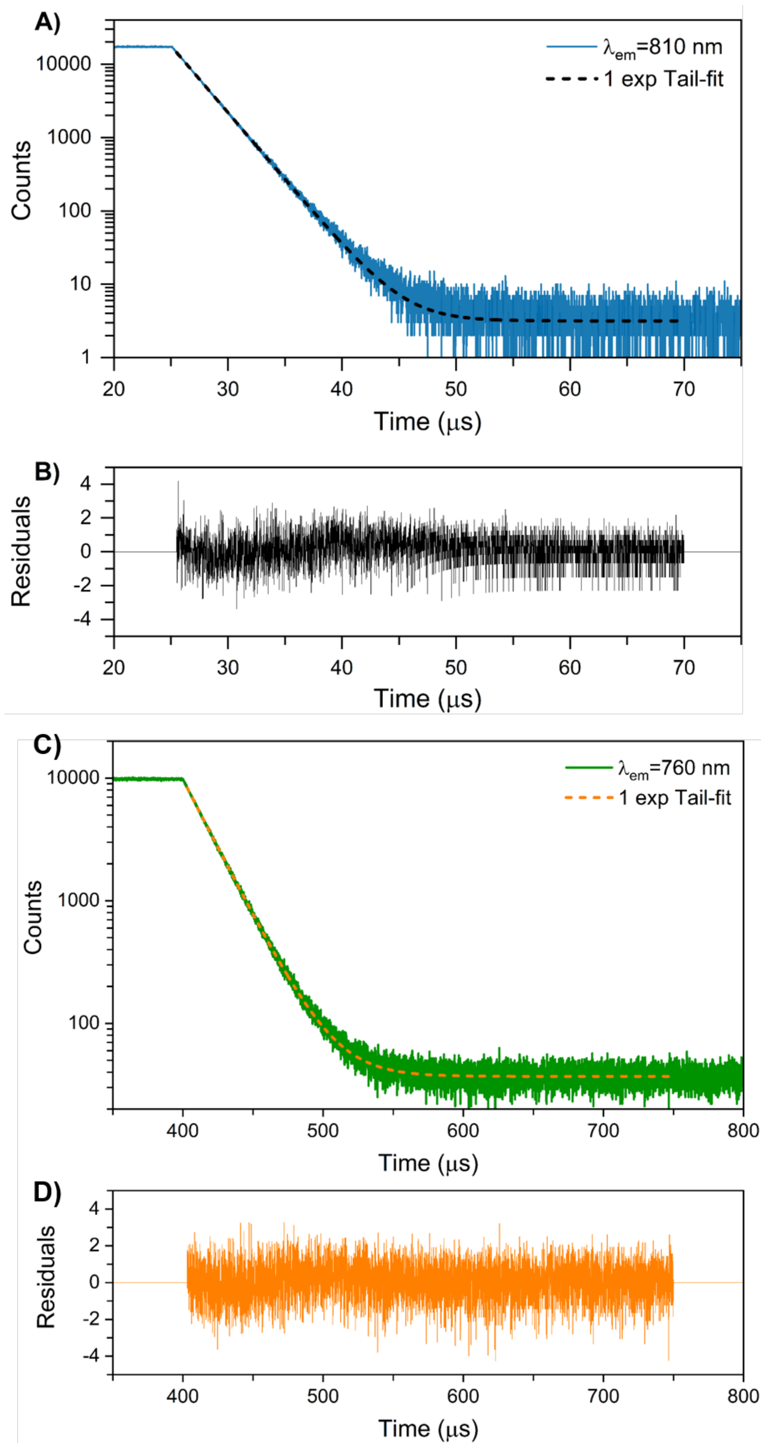

Figure S15: Mono-exponential tail-fit models and corresponding residuals of decay curves reported in Figure S14. **A)** and **B)** refer to 800-Ag_N-DNA (A_1=14399.0 ± 78.5 counts; $\tau_{1=}$2.385 ± 0.009 μs; χ^2=0.917), whereas **C)** and **D)** are related to 760-Ag_N-DNA (A_1=8278.0 ± 61.2 counts; $\tau_{1=}$19.456 ± 0.109 μs; χ^2=0.993).

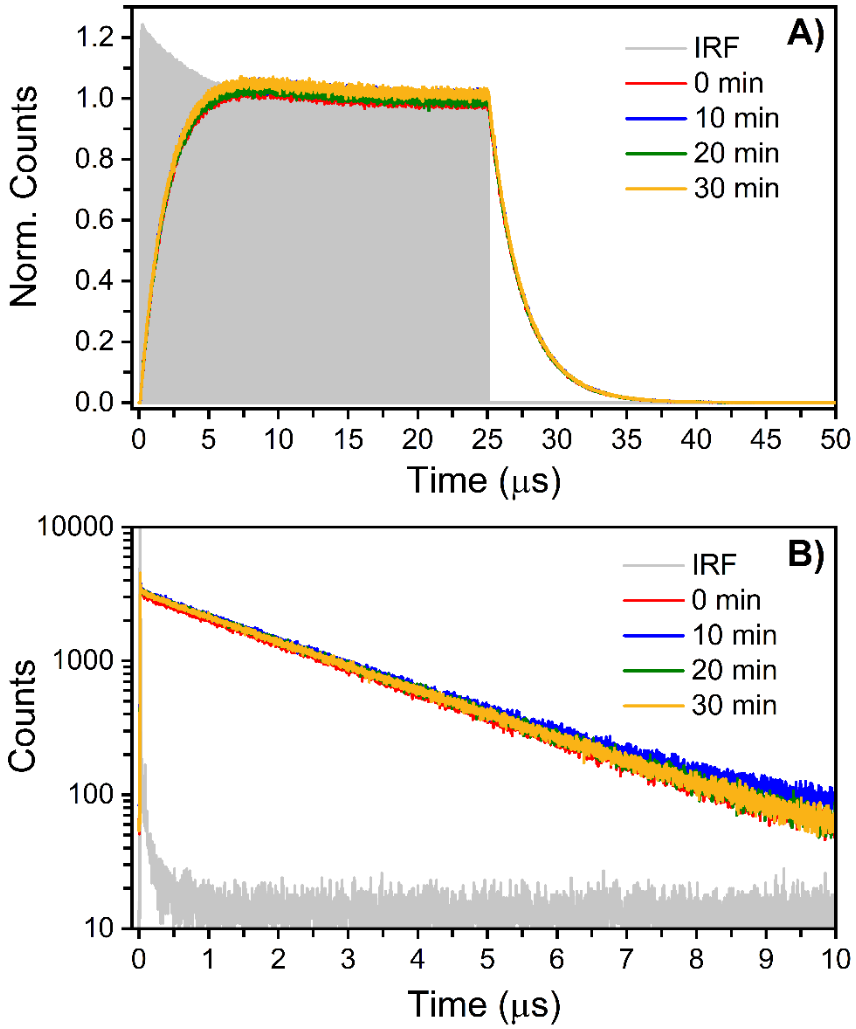

Figure S16: 800-Ag$_N$-DNA in 10 mM NH_4OAc at λ_{em}=810 nm (λ_{exc}=374.3 nm) measured at T=25 °C with **A)** burst mode (normalized) and **B)** TCSPC mode. The IRF traces are reported in light gray. 0 min represents the sample prior to oxygen removal, while 10, 20 and 30 min refer to the time spent removing the oxygen dissolved in solution with the help of a water jet pump. Corresponding decay times are reported in Table S3.

Table S3: Luminescence decay time values for 800-Ag$_N$-DNA in 10 mM NH_4OAc at λ_{em}=810 nm (λ_{exc}=374.3 nm) measured at T=25 ºC. Both burst mode and TCSPC decay curves were tail-fitted with mono-exponential functions.

	**Burst Mode**			**TCSPC**		
**Time (min)**	**A_1 (Counts)**	**τ_1 (μs)**	**χ^2**	**A_1 (Counts)**	**τ_1 (μs)**	**χ^2**
0	14399.0 ± 78.5	2.385 ± 0.009	0.917	2813.6 ± 17.7	2.349 ± 0.012	1.021
10	12286.4 ± 72.9	2.390 ± 0.010	0.939	3012.0 ± 18.6	2.339 ± 0.012	1.028
20	12197.5 ± 73.0	2.390 ± 0.010	0.948	2984.9 ± 18.3	2.344 ± 0.012	1.037
30	12171.6 ± 71.5	2.392 ± 0.010	0.913	2963.7 ± 17.7	2.341 ± 0.011	0.977

3. References:

(1) O'Neill, P. R.; Gwinn, E. G.; Fygenson, D. K. UV Excitation of DNA Stabilized Ag Cluster Fluorescence via the DNA Bases. *J. Phys. Chem. C* **2011**, *115* (49), 24061–24066. https://doi.org/10.1021/jp206110r.

(2) Lakowicz, J. R. *Principles of Fluorescence Spectroscopy*; Springer Science & Business Media, 2006. https://doi.org/10.1007/978-0-387-46312-4.

(3) Bogh, S. A.; Carro-Temboury, M. R.; Cerretani, C.; Swasey, S. M.; Copp, S. M.; Gwinn, E. G.; Vosch, T. Unusually Large Stokes Shift for a Near-Infrared Emitting DNA-Stabilized Silver Nanocluster. *Methods Appl. Fluoresc.* **2018**, *6* (2), 024004. https://doi.org/10.1088/2050-6120/aaa8bc.

(4) Brouwer, A. M. Standards for Photoluminescence Quantum Yield Measurements in Solution (IUPAC Technical Report). *Pure Appl. Chem.* **2011**, *83* (12), 2213–2228. https://doi.org/10.1351/PAC-REP-10-09-31/MACHINEREADABLECITATION/RIS.

(5) Liisberg, M. B.; Kardar, Z. S.; Copp, S. M.; Cerretani, C.; Vosch, T. Single-Molecule Detection of DNA-Stabilized Silver Nanoclusters Emitting at the NIR I/II Border. *J. Phys. Chem. Lett.* **2021**, *12* (4), 1150–1154. https://doi.org/10.1021/ACS.JPCLETT.0C03688.

(6) Gonzàlez-Rosell, A.; Cerretani, C.; Mastracco, P.; Vosch, T.; Copp, S. M. Structure and Luminescence of DNA-Templated Silver Clusters. *Nanoscale Adv.* **2021**, *3* (5), 1230–1260. https://doi.org/10.1039/d0na01005g.

(7) Schultz, D.; Gardner, K.; Oemrawsingh, S. S. R.; Markešević, N.; Olsson, K.; Debord, M.; Bouwmeester, D.; Gwinn, E. Evidence for Rod-Shaped DNA-Stabilized Silver Nanocluster Emitters. *Adv. Mater.* **2013**, *25* (20), 2797–2803. https://doi.org/10.1002/adma.201204624.

(8) Liisberg, M. B.; Krause, S.; Cerretani, C.; Vosch, T. Probing Emission of a DNA-Stabilized Silver Nanocluster from the Sub-Nanosecond to Millisecond Timescale in a Single Measurement. *Chem. Sci.* **2022**, *13* (19), 5582–5587. https://doi.org/10.1039/D2SC01137A.
